# Predictors of Biracial adolescent racial self‐categorization when confronted with monoracist demographic forms

**DOI:** 10.1111/jora.70012

**Published:** 2025-02-24

**Authors:** Victoria Vezaldenos, Deborah Rivas‐Drake, David R. Schaefer, Adriana J. Umaña‐Taylor, Sara I. Villalta, Bernardette Pinetta

**Affiliations:** ^1^ University of Michigan Ann Arbor Michigan USA; ^2^ University of California Irvine California USA; ^3^ Harvard University Cambridge Massachusetts USA; ^4^ Loyola Marymount University Los Angeles California USA; ^5^ University of California Los Angeles California USA

**Keywords:** biracial, ethnic‐racial identity, logistic regression, racial self‐categorization

## Abstract

The current study draws from literature on Multiracial ethnic‐racial identity development processes and utilizes logistic regression models to identify what factors inform ethnic‐racial self‐categorization choices when confronted with a monoracial paradigm of race in a sample of Biracial high school students. Separate logistic regression models analyzed how family ethnic‐racial socialization, phenotype, friend groups, and experiences with discrimination are associated with the racial category for Biracial White, Asian, Black, Native American, and Latinx youth, respectively, when asked to choose just one racial background. Results suggest that the associations of family ethnic‐racial socialization, experiences with discrimination, and skin color with self‐categorization vary in directionality and strength for different groups of Biracial adolescents. However, adolescents with a greater proportion of friends in a given ethnic‐racial group were more likely to self‐categorize with that respective ethnic‐racial group across all models. These findings provide a nuanced understanding of how Biracial youth draw on various aspects of their lived experiences when confronting monoracism.

## INTRODUCTION

It is estimated that by 2050, 20% of Americans will belong to two or more racial categories (Perez & Hirschman, 2009; Talbot, 2008). The rapid growth of the Multiracial^1^ population in the United States (U.S.) is sometimes thought to indicate that our society is becoming one in which racial categories are arbitrary and no longer meaningful (Root, 2003; see footnotes). Along with these misconceptions, there is a paucity of literature analyzing ethnic‐racial identity development for Multiracial people; meanwhile, similar work examining these processes in monoracial people has continued to flourish (e.g., Rivas‐Drake et al., 2014; Umaña‐Taylor et al., 2014). Empirical work that has centered on Multiracial perspectives demonstrates that socially constructed racial categories, reified as a result of dominant monoracist epistemologies, continue to have significant implications on the development of Multiracial youth (Harris, 2016; Rockquemore & Brunsma, 2002).

Monoracism refers to the systemic oppression of Multiracial individuals through the enforcement of monoracial paradigms of race (Gabriel et al., 2023; Harris, 2016; Johnston & Nadal, 2010). In a sociopolitical context that emphasizes monoracial group identification, Multiracial people are often forced to pick between their identities across situational contexts–this is known as a racial category decision (Campbell, 2003, 2007; Gabriel et al., 2023; Harris, 2016, 2017; Johnston & Nadal, 2010; Rockquemore et al., 2009). These frequent requests to foreground a singular racial category in order to conform to a monoracial paradigm are one of the reasons why it may be difficult to have a bridged, Multiracial identity (Gabriel et al., 2023; Harris, 2016; Johnston‐Guerrero et al., 2020). In fact, we see a higher prevalence of poor developmental and psychosocial outcomes in the population of mixed‐race youth, presumably due to challenges in navigating and resolving their ethnic‐racial identities within contexts that privilege singular, mutually exclusive racial categories (AhnAllen et al., 2006; Bradshaw, 1992; Lorenzo‐Blanco et al., 2013; Nishina & Witkow, 2020; Rivas‐Drake et al., 2014; Root, 2003). Yet, less is known about what interpersonal and social factors Multiracial youth might draw on when confronting such monoracist paradigms.

Furthermore, much of the ethnic‐racial identity development research omits Multiracial participants altogether or lumps them into an all‐encompassing “Multiracial” category; this leads to a lack of precision as the processes under investigation may differ considerably across subgroups of Multiracial youth (e.g., Biracial Black youth in comparison to Biracial Latinx youth; Charmaraman et al., 2014; Nishina & Witkow, 2020). This is problematic, as other studies have begun to reveal significant variation between Multiracial individuals with different racial backgrounds, pointing to the necessity for identity scholars to explore such within‐group differences (Christophe et al., 2022; Haydel et al., 2023; Rockquemore & Brunsma, 2002; Root, 2003; Townsend et al., 2012).

In the current study, we explore contextual factors that may inform racial category decisions across different groups of Biracial youth (Biracial Black, Biracial Latinx, etc.) when confronted with a monoracial paradigm of race. Rather than treating Biracial youth as a monolithic category, we disaggregate a sample of Biracial adolescents into subgroups to reveal potential within‐group variations (Charmaraman et al., 2014; Nishina & Witkow, 2020). Knowledge of how racial category decisions differ among Biracial youth according to specific racial group membership can be leveraged to create contexts that support Multiracial youth development within the confines of a monoracist society, thus improving their psychological adjustment and quality of life.

## BACKGROUND

### Biracial identity development

Multiracial^2^ identity development theory conceptualizes the social construction of race as containing three distinct facets: *racial identification*, *racial identity*, and *racial category* (Gabriel et al., 2023; Rockquemore et al., 2009). *Racial identification* refers to external appraisals of one's ethnic‐racial background whereas *racial identity* relates to a Multiracial person's internal meaning‐making of their ethnic‐racial heritages. *Racial category* (i.e., self‐categorization), on the other hand, is how a Multiracial person might identify themselves across time and context as constrained by the options available to them. The options available to Multiracial youth might be in discordance and fluctuate across time and context (Gabriel et al., 2023; Rockquemore et al., 2009; Zamora & Padilla, 2024) and thus change how they respond to questions of self‐categorization (Mauer et al., 2020; Phinney & Alipuria, 1996). Multiracial people might adjust their racial category selection(s) based on their varied life experiences and psychological resolution of critical moments that convey messages about ethnic‐racial group membership (Bradshaw, 1992; Campbell, 2007; Kich, 1992; Miville et al., 2005; Root, 1990, 2003).

Critical moments are experiences that result in internal or external conflict, ultimately informing Multiracial people's clarity about their identities. These experiences may include difficulty assimilating to monoracial in‐groups, talking with monoracial family members about race, being a target of monoracial microaggressions, and the like (Gabriel et al., 2023; Nadal et al., 2013). Multiracial youth engage in sensemaking of these moments and incorporate them into their ethnic‐racial self‐concept, thus informing their self‐categorization choices (Bradshaw, 1992; Kich, 1992; Rockquemore et al., 2009; Root, 1990).

When encountering opportunities for self‐categorization (e.g., applying to college, filling out surveys, completing health forms), Multiracial people are often forced to select just one ethnic‐racial category to identify with. This monoracist framing reduces the complexity of Multiracial identities and forces assimilation to discrete monoracial categories (Gabriel et al., 2023; Harris, 2016). The racial category one selects might vary across time and contexts depending on how they have reconciled the aforementioned critical moments (Harris, 2016; Miville et al., 2005; Rockquemore et al., 2009) and how the question is asked (Mauer et al., 2020; Phinney & Alipuria, 1996). As Multiracial youth develop within a monoracist society, they will likely be confronted with these forced‐choice questions from a very young age. However, encountering such questions during adolescence is particularly relevant for identity development.

#### Developmental considerations

Adolescence is a critical developmental period for Biracial youth, as they strive to form supportive peer relationships, navigate in‐groups and out‐groups, develop an independent self‐concept distinct from their family context, and grapple with experiences with discrimination (Doyle & Kao, 2007a; Miville et al., 2005; Rivas‐Drake et al., 2014; Rivas‐Drake & Umaña‐Taylor, 2019; Root, 1990). As teens critically reflect on racial identification cues from family, peers, community, and their environment, they assimilate these messages into their self‐concept (Miville et al., 2005; Root, 1990). These meaningful adolescent experiences ultimately inform youth's understanding of their ethnic‐racial identity and, as a result, their racial category selections (Bradshaw, 1992; Gabriel et al., 2023; Rockquemore et al., 2009; Root, 1990). Thus, when students are asked to self‐categorize themselves racially, they may be drawing from several experiences to determine how to report their racial group membership. In this study, we sought to explore factors that contribute to Biracial high school students' ethnic‐racial categorization, operationalized as students' self‐selected racial category when confronted with a monoracist paradigm of race–namely, being asked to choose which monoracial group they most identify with.

Although there is a dearth of attention to developmental differences *within* Multiracial populations, we can turn to systematic reviews of associations of ethnic‐racial socialization with ethnic‐racial identity beliefs among diverse youth, including Multiracial youth. Such reviews indicate that the effect sizes are stronger among high school than elementary and middle school samples (Huguley et al., 2019). That said, Biracial and Multiracial youth often have earlier exposure than monoracial youth to out‐group members, both within and beyond their family context, and they experience a lack of representation in peer groups and schools. These kinds of experiences may accelerate Multiracial adolescents' capacity for making nuanced racial category choices (Nishina & Witkow, 2020). For example, the need to navigate conflicting (i.e., affirming and invalidating) messages about multiple ethnic‐racial groups to which Multiracial people belong emerges across the lifespan and may be salient across developmental periods (e.g., Jones & Rogers, 2022; Kellogg & Liddell, 2012; Museus et al., 2016). This may be why, at times, ethnic‐racial socialization, phenotypic (mis)categorization, and discrimination experiences inform racial understandings (including self‐categorization) of Multiracial youth in adolescence (e.g., Jones & Rogers, 2022) and young adulthood (e.g., Does et al., 2023; Kellogg & Liddell, 2012; Museus et al., 2016) in similar ways.

### Racial category: The forced‐choice question

Only recently have scholars had access to large enough datasets allowing them to explore Multiracial adolescent *racial identity* and *identification* quantitatively (Csizmadia, 2011; Doyle & Kao, 2007b; Echols et al., 2018; Johnston et al., 2014; Morning & Saperstein, 2018; Rockquemore et al., 2009; Saperstein et al., 2016; Song et al., 2022). Although *racial category* has been explored as an antecedent of adjustment outcomes, less is known about what individual and contextual factors might predict a Multiracial individual's racial category choices (Campbell, 2003, 2007; Gabriel et al., 2023; Herman, 2001). Learning more about the experiences that inform these decisions might support practitioners' capacities to create contexts that affirm Multiracial youth development within a monoracist society (Harris, 2016; Johnston & Nadal, 2010).

Forcing Biracial people to choose one of their racial identities over others is complex and can itself present a critical moment that results in self‐reflection about what these identities mean to them (Bradshaw, 1992; Miville et al., 2005). Root (1990, 2003) articulates how Multiracial people exert both mental and emotional effort when forced to select one identity. Multiracial people might feel that they must decide between selecting a response that aligns with the ethnic‐racial group they feel closest to or the ethnic‐racial group others perceive them to be, which is not always congruent. This invalidation of their multiple identities is, unfortunately, a common experience that merits further exploration. As a means to better critique such monoracist paradigms, research is needed on what assets Multiracial people draw on when confronting such monoracism (Harris, 2016; Johnston & Nadal, 2010).

When Biracial youth are forced to choose between their multiple racial identities on demographic forms, they may ultimately select one of their ethnic‐racial categories to report, or they might refuse the question by skipping it entirely or writing in a response that asserts both of their identities. Respondents that select just one identity when forced to choose may be displaying a *protean identity* status–fluidly selecting a single identity that seems most appropriate given the context (Gabriel et al., 2023; Rockquemore, 1998; Rockquemore & Brunsma, 2002; see also Daniel (1996) “integrative identities” and Choi‐Misailidis (2004) “singular identity”). Whereas respondents that assert their Biracial identity may be displaying a *border identity* status in which they exclusively identify as Biracial, Multiracial, or mixed race (Gabriel et al., 2023; Rockquemore, 1998; Rockquemore & Brunsma, 2002; see also Choi‐Misailidis (2004)'s “integrated identities”). Such identity typologies have been studied extensively in Biracial Black‐White populations but still merit exploration in other Multiracial groups (Brunsma, 2005; Herman, 2004; Lee & Bean, 2004; Rockquemore, 1998; Rockquemore et al., 2009; Wright et al., 2003).

Further, the field has called on scholars to consider how racial identity, racial category, and racial identification converge and diverge for Multiraical youth while also considering the contextual factors that influence these identity development processes (Gabriel et al., 2023; Rockquemore et al., 2009). We aim to explore these relationships in the current study. There are many potential antecedents of racial category for Biracial adolescents in the literature, yet few have been explored empirically among youth (Yoo et al., 2016); some of the most important are phenotype, experiences with discrimination, family ethnic‐racial socialization, and racial composition of peer groups.

#### The role of phenotype and skin color

Phenotype, or the visual representation of one's genes, has a significant influence on the ethnic‐racial category choices for Multiracial people. Biracial individuals are often racially identified (and misidentified) by others based on how they phenotypically present (Albuja, Gaither, et al., 2019; Gabriel et al., 2023; Pauker et al., 2018; Rockquemore et al., 2009), and they may often settle on this identity assigned by society (Gabriel et al., 2023; Miller, 2020; Rockquemore & Brunsma, 2002; Root, 1990, 2003; Sims, 2016; Talbot, 2008). Extensive literature has documented that Multiracial people garner validation of their ethnic‐racial identity from monoracial others within their in‐groups (Brown, 1997; Franco, 2019; Gabriel et al., 2023; Miller, 2020; Pauker et al., 2018; Root, 1990; Sims, 2016; Talbot, 2008; Thekkedam, 2013). This validation sometimes comes from how they are racially perceived, with skin color being a key indicator of racial group membership (AhnAllen et al., 2006; Franco et al., 2016; Rockquemore et al., 2009; Rockquemore & Brunsma, 2002). Research has demonstrated that the racial identification that others assign to a Multiracial person often influences them in choosing to self‐categorize as that same identity (AhnAllen et al., 2006; Gabriel et al., 2023; Gaither, 2015; Rockquemore & Brunsma, 2002).

Historically, Multiracial people have had less choice in the matter as they have been subjected to hypodescent, or the one‐drop rule. This stipulated that Multiracial people with any sort of African ancestry must identify as Black. As a result, Black‐White Biracial people have been systematically excluded from the privilege of claiming a White identity (Brown, 1997; Miller, 2020; Thekkedam, 2013). Although no longer legally enforced, the rule of hypodescent is still informally imposed socially, such that Biracial White people are often visually perceived to be, and thus classified as, members of their minoritized ethnic‐racial group (Albuja, Sanchez, et al., 2019; Chen et al., 2014, 2018; Ho et al., 2011; Miller, 2020; Sims, 2016; Thekkedam, 2013; Young et al., 2021). Yet the opposite is often true for Multiracial Native Americans, who have historically been encouraged to assimilate to whiteness, a concept known as hyperdescent (Doyle & Kao, 2007b; Gullickson & Morning, 2011). Scholars have since explored when, why, and how Multiracial people claim monoracial identities and what social cues aid in a Multiracial person's ability to pass as a member of a monoracial group. Oftentimes this is determined by phenotype, in that a young person might feel they are able to claim the monoracial ethnic‐racial identity for the group they most closely resemble, or are perceived to be (Gabriel et al., 2023; Gaither, 2015; Miville et al., 2005; Rockquemore et al., 2009; Sims, 2016).

The possibilities for claiming different ethnic‐racial identities are linked to the privilege of phenotypic proximity to whiteness. For example, some Biracial individuals who are White‐presenting may elect to pass as White to gain acceptance in predominantly White spaces; such passing ultimately results in fewer experiences with discrimination across the lifespan (Albuja et al., 2018; Bradshaw, 1992; Ho et al., 2011; Thekkedam, 2013). Others make an intentional choice *not* to pass as White, despite being White‐presenting. Most often, Biracial Black people are the least proximal to Whiteness. This is because Biracial Black people typically carry phenotypic features that create a more salient Black ethnic‐racial identity and are othered as non‐White in social contexts. Biracial Black individuals are labeled as monoracial Black by others more frequently than other more phenotypically ambiguous individuals; they also report increased stress and experiences with discrimination (Albuja, Gaither, et al., 2019; Albuja, Sanchez, et al., 2019; Bradshaw, 1992; Brown, 1997; Gaither, 2015; Ho et al., 2011; Thekkedam, 2013).

Although an individual's racial phenotype is made up of multiple features, including hair texture and color, eye shape and color, and nose and lip shape, research has shown skin color to be the primary characteristic used to classify a person's race (Brown Jr. et al., 1998; Feliciano, 2016). For example, one study used observers' assessments of photos of White, Black, Latino, and Multiracial individuals to examine how phenotypic markers influenced racial categorization. Findings revealed skin color to be the strongest indicator used to categorize individuals by race, with light skin being associated with whiteness, medium‐colored skin with Latinidad, and dark skin with Blackness. In sum, both Biracial and monoracial people are racially categorized by others based on how they phenotypically present–a categorization process chiefly influenced by skin color–and may in turn select the racial category for whatever monoracial identity is frequently assigned to them (Gabriel et al., 2023; Gaither, 2015; Miller, 2020; Root, 1990, 2003; Sims, 2016; Talbot, 2008).

#### Racial category choices informed by experiences with discrimination

Miville et al. (2005) explain that acts of discrimination are often perceived by Multiracial people as society imposing a racialized identity onto them that is distinct from the subjective Multiracial identities they hold. Multiracial people who experience monoracial forms of discrimination may internalize these messages and incorporate them into their self‐concept, resulting in a reference group orientation towards the imposed identity (Franco et al., 2016; Johnston & Nadal, 2010; Root, 1990; sometimes referred to as “pull factors”). Having a racial identity reinforced due to experiences with discrimination can make the identity more salient, as people in youths' environments have repeatedly perceived them to be a member of a particular ethnic‐racial group and have discriminated against them in response (Christophe et al., 2022; Franco et al., 2016; Miville et al., 2005; Root, 1990, 2003).

Informed by previous work, the current study included measures of both peer and institutional discrimination to predict racial category choices among Biracial adolescents. Biracial people that experience institutional forms of discrimination may feel that they are being situated within a monoracial paradigm and a singular racial identity is being forced upon them (Christophe et al., 2022; Franco et al., 2021; Johnston & Nadal, 2010; Miville et al., 2005; Root, 1990; Yoo et al., 2016). This may result in feeling like one needs to claim the ethnic‐racial identity that serves as the basis for the experienced discrimination (e.g., anti‐Blackness reinforcing a Black ethnic‐racial identity; Davenport et al., 2022; Franco et al., 2016; Gullickson & Morning, 2011). This relationship is slightly complicated when acts of discrimination come from in‐group peers. If discrimination stems from a member of an adolescent's in‐group (those that they do share a racial identity with), youth may instead feel that they do not belong, potentially making the identity less salient (Franco, 2019; Rockquemore & Brunsma, 2002; Root, 1998; sometimes referred to as “push factors”). For instance, Multiracial youth may be told they are not authentic enough to be a part of a monoracial space by in‐group members, which may cause youth to turn away from or reject this identity (Franco, 2019; Museus et al., 2016; Norman et al., 2023).

#### Family ethnic‐racial socialization can inform racial identity

The socialization that youth receive at home about race and ethnicity is also a critical context for ethnic‐racial identity development. Several studies have noted the importance of the home environment and how explicit conversations about race with parents or siblings (overt ethnic‐racial socialization) can aid in healthy identity development among Multiracial people (Atkin & Jackson, 2021; Bradshaw, 1992; Christophe et al., 2021; Green et al., 2021; Green & Bryant, 2023a; Jackson et al., 2019; Renn, 2000; Talbot, 2008). Additionally, cultural practices and knowledge instilled within the home (covert ethnic‐racial socialization) can support Multiracial people's understanding of their group memberships, particularly among those who are phenotypically ambiguous (AhnAllen et al., 2006; Atkin, Jackson, et al., 2022; Csizmadia & Atkin, 2022; Green et al., 2021; Green & Bryant, 2023a; Miville et al., 2005; Renn, 2000).

Overt conversations about what it means to be a member of one's ethnic‐racial group can encourage youth to think critically about their identities and assimilate them to their self‐concept (Atkin & Jackson, 2021; Atkin, Jackson, et al., 2022; Bradshaw, 1992; Csizmadia & Atkin, 2022; Green et al., 2021; Green & Bryant, 2023a; Talbot, 2008). However, empirical research has shown that this can be a complicated process for Multiracial youth, as they are often born of interracial marriages in which parents have differing cultural practices (Atkin & Yoo, 2019; Christophe et al., 2021; Green & Bryant, 2023b; Lorenzo‐Blanco et al., 2013; Miller, 2020). Biracial home environments may or may not emphasize both cultures equally, and conversations about race may be complicated for parents to navigate due to their incongruent ethnic‐racial experiences. Further, the children of interracial relationships obtain a new, Multiracial identity that neither parent is typically equipped to support and unpack, as this identity itself entails a unique lived experience (Atkin & Jackson, 2021; Atkin, Jackson, et al., 2022; Atkin & Yoo, 2019; Green & Bryant, 2023a, 2023b; Jackson et al., 2019; Lorenzo‐Blanco et al., 2013; Miller, 2020).

Within the home environment, Multiracial youth may be covertly socialized through exposure to diverse languages, religions, music, food, cultural practices, and beliefs, all of which may inform their ethnic‐racial identity and, thus, their resulting racial category selections. Parents may also introduce blended cultural practices and ideals at home, giving adolescents cultural knowledge from both ethnic‐racial groups. However, if one or both parents are absent, if there is racism or other tensions expressed between families, or if caregiving support from one ethnic‐racial side of the family is greater than the other, adolescents may have differential exposure to these cultural practices and norms (AhnAllen et al., 2006; Atkin & Yoo, 2019; Green & Bryant, 2023a; Miller, 2020; Miville et al., 2005). As a result, Multiracial adolescents may develop a strong reference group orientation to only one ethnic‐racial group and then may choose to self‐categorize as a member of this group when forced to pick just one ethnic‐racial identity option. For these reasons, both overt and covert ethnic‐racial socialization were included as potential predictors of racial self‐categorization in this study.

#### The potential role of racial composition of friend groups

Prior literature has shown that peers play an important role in the identity development processes during adolescence (e.g., Rivas‐Drake et al., 2017), but less is known about how friendships inform ethnic‐racial identity choices for Multiracial adolescents specifically (Campbell, 2007; Renn, 2000; Root, 1990; Yoo et al., 2016). Research has shown that peer group changes can lead to changes in ethnic‐racial identity choices for Multiracial youth (Echols et al., 2018) and that once Multiracial youth gain membership to an in‐group, this peer acceptance can validate and reinforce their identity (Doyle & Kao, 2007a; Franco, 2019; Rivas‐Drake et al., 2014; Rivas‐Drake & Umaña‐Taylor, 2019; Sims, 2016).

While scholarship on Multiracial adolescents and young adults has theorized that racially homogenous social networks would relate to self‐categorizing as a singular racial identity (another “pull factor”), this has yet to be tested empirically (Rockquemore, 1998; Rockquemore & Brunsma, 2002; Rockquemore et al., 2009). Biracial adolescents who affiliate with a high concentration of friends of a given ethnic‐racial group may be more inclined to identify with this group. Consequently, we used the proportion of youth's friends in a given ethnic‐racial category to predict youth's own membership in that category (e.g., does the proportion of Latinx friends predict Latinx self‐categorization among Biracial Latinx youth).

### The current study

As informed by the Model of Multiracial Racialization (Gabriel et al., 2023), we explored how both individual characteristics and interpersonal experiences might predict Biracial high school students’ self‐selected *racial category* while also controlling for contextual factors like geographic location. Focusing on Biracial youth with multiple possible racial category choices, we examined how phenotype, experiences of discrimination, familial ethnic‐racial socialization, and friends from the focal ethnic‐racial category relate to their racial category choices when confronted with a monoracist paradigm of race (being asked to pick just one ethnic‐racial group that they felt closest to; Harris, 2016; Johnston & Nadal, 2010).

Following the literature reviewed, we expected that youth would be more likely to self‐categorize with a racially minoritized group if they had higher levels of family ethnic‐racial socialization (both overt and covert), more experiences with discrimination (both peer and institutional), and a higher proportion of friends in that racially minoritized group. Additionally, we hypothesized that youth with darker skin would likely have lived experiences that informed their forced‐choice response such that they would be more likely to self‐categorize with a racially minoritized group; conversely, we expected that youth with lighter skin might be less likely to self‐categorize with a racially minoritized group when presented with a forced‐choice situation (e.g., Bradshaw, 1992; Ho et al., 2011; Root, 2003; Thekkedam, 2013).

In regard to selecting a White identity, we posited that there would be an inverse relationship with discrimination experiences, such that lower levels of both peer and institutional discrimination would predict a higher likelihood of selecting White as their forced choice racial category. Following the same argument as for Biracial non‐White youth, we predicted that for Biracial White youth, a higher proportion of friends that also self‐categorize as White would increase the likelihood that a Biracial person claims a White identity in their forced‐choice response. As informed by the literature on phenotype, hypodescent, and hyperdescent (Doyle & Kao, 2007b; Gullickson & Morning, 2011; Harris & Sim, 2002; Ho et al., 2011; Root, 2003; Thekkedam, 2013), we hypothesized that Biracial Black‐White students would have the least proximity to whiteness and therefore would be the least likely to claim a monoracial White identity and expected the converse for Biracial Native American‐White students compared to other Biracial White students (Campbell, 2003, 2007).

In addition, although some scholars have asserted that an ideal identity for Biracial people is one that incorporates both of their ethnic‐racial identities, little is known about what factors inform or support this choice (Root, 2003). There is no research to date that has explored these mechanisms quantitatively, particularly with diverse subgroups of Multiracial youth (Gabriel et al., 2023). Therefore, we offer an analysis of youth who intentionally assert a Biracial or *Border identity* (i.e., those who refuse to answer the forced choice question; Rockquemore, 1998; Rockquemore & Brunsma, 2002; see also Daniel (1996) “blended identity”) that is largely exploratory, with no specific hypotheses.

## METHOD

### Participants and procedure

Data for the study were drawn from the Teen Identity Development and Education Study (TIDES), which is a project examining the ethnic‐racial identity and peer relationships over time among ethnically and racially diverse adolescents. TIDES data were collected at two high school sites, sampling 9th to 12th grade students in the Midwest and Southwest regions of the U.S. between 2017 and 2018. The sites were selected based on similarities in student demographics, level of ethnic‐racial diversity, and measures of academic achievement. A passive parental consent procedure was employed whereby parents were asked to indicate their desire for their student to opt out of the survey; the entire student body was invited to participate, and students were granted approximately 50 min of class time to complete the self‐report survey. The study was approved by the IRBs at the principal investigators' institutions.

The larger TIDES study included three waves of data collection; however, the present analysis draws from wave 2 data (fall semester of the 2017–2018 academic year), as it contains the largest number of Biracial students. The Biracial subset was identified based on responses to a survey question that asked students to select all ethnic backgrounds that applied to them. Specifically, of the 3562 participants with valid race data at this wave, 811 selected exactly two ethnic‐racial categories (with 21 unique racial categorization combinations). Of these, 697 Biracial respondents had complete data for all variables. Using this analytic sample, we then created subsamples of biracial students, each based on one racial category (e.g., Biracial Asian, Biracial Black, etc.). See Table 1 for the tabulation of each Biracial group of students and their forced‐choice responses. Each Biracial student was placed into two subsamples for our statistical analysis (i.e., a Black‐White student was placed into both the Biracial Black and Biracial White subsamples). Refer to Table 2 for complete descriptive statistics regarding the analytic sample of Biracial students.

**TABLE 1:** Tabulation of Biracial students and their forced choice responses.

	Freq.	Percent	Cum.
Biracial student identities			
Biracial American Indian/Native American	147	21.09	21.09
Biracial Asian	131	18.79	39.88
Biracial Black/African American	263	37.73	77.61
Biracial Latino/Hispanic	334	47.92	125.53
Biracial White	472	67.72	193.25
Biracial Middle Eastern	23	3.30	196.55
Biracial Other	14	2.01	198.56
Biracial Pacific Islander	10	1.44	200.00
Total	1394	200.00^a^	
Forced choice responses			
American Indian/Native American	28	4.02	4.02
Asian/Pacific Islander	49	7.03	11.05
Black/African American	192	27.55	38.6
Latino/Hispanic	145	20.80	59.4
White	213	30.56	89.96
Middle Eastern	8	1.15	91.11
Other	10	1.43	92.54
Biracial^b^	52	7.46	100.00
Total	697	100.00	

^a^ The total sample of Biracial students with complete cases is 697. However, because students are Biracial, they are counted twice in the tabulations, resulting in a cumulative percent of 200.

^b^ Students who refused the forced choice response were coded as asserting a Biracial identity.

**TABLE 2:** Sample descriptive statistics.

	Freq.	Percent	Cum.
Gender			
Girl	369	52.94	52.94
Boy	318	45.62	98.57
Genderqueer/non‐binary^a^	10	1.43	100.00
Total	697	100.00	
Location			
Southwest	503	72.17	72.17
Midwest	194	27.83	100.00
Total	697	100.00	
Grade			
9th	245	35.15	35.15
10th	159	22.81	57.96
11th	172	24.68	82.64
12th	121	17.36	100.00
Total	697	100.00	
Student's generational immigration status^b^			
1st generation	22	3.16	3.16
2nd generation	196	28.12	31.28
3rd generation	159	22.81	54.09
4th + generation	320	45.91	100.00
Total	697	100.00	

^a^ Students chose to write in a variety of different gender identities that were outside of the girl/boy gender binary. We collapsed these youth to create an all‐encompassing genderqueer/non‐binary category, although the youth respondents may not have used these exact terms to describe themselves.

^b^ The control variable for immigration generation status was created using students' reports of whether they, their parents, and/or their grandparents were born in the U.S., on U.S. territories, on U.S. military bases, or abroad. More details regarding how this was coded are offered on pages 19 & 20.

### Measures

#### Racial category

In the demographic section of the TIDES survey, students were asked to select all ethnic‐racial groups with which they identified. Students who selected more than one ethnic‐racial group were then confronted with a monoracist question.^3^ They were asked to select which ethnic‐racial identity they “feel most a part of.” Their answer to this question is referred to as their forced‐choice response and, in the present study, conceptualized as youths' self‐selected *racial category*. The students were asked to pick just one of their identities, and therefore in the moment, they were determining how to identify themselves given the constrained options available to them.

If the students checked multiple responses for the “all that apply” question but did not answer the forced‐choice question, they were coded as having chosen a Multiracial identity rather than selecting just one of the groups.^4^ We conceptualized this as the student asserting a *border identity* as they explicitly chose to exclusively identify as Biracial rather than selecting just one ethnic‐racial category. However, if a student selected just one of the groups when presented with the forced‐choice option, a variable was created to indicate that choice. For example, a Biracial Asian student who selected Asian as their forced‐choice response rather than selecting any other identity was coded accordingly (i.e., selection of Asian as a forced‐choice option = 1; not selecting = 0). We did this for every option available (see Table 1 for a complete tabulation of Biracial student forced‐choice responses). These dichotomized forced‐choice responses serve as the outcomes of interest in this study.

#### Phenotype (skin color)

Phenotype data were coded utilizing digitized yearbook photos of students from both school sites. Research assistants coded the yearbook photos to assess skin color, hair texture, nose, and lip shape. This study utilized the researchers' assessments of student skin color as coded according to the NIS Skin Color scale (Massey & Martin, 2003). The NIS Skin Color Scale includes 10 numbered hands, with 1 being the lightest and 10 the darkest. When coding the student yearbook photos, researchers viewed the digitized images and the skin color scale in split screen mode to assess the best match while controlling for possible differences in lighting across coders' monitors. No less than three raters assessed each students' skin color. Mean scores across raters were used in the analysis. The pool of raters comprised 25 individuals who identified as female, 4 as male, and 1 who identified as being of some “other” gender. Forty‐three percent of raters identified as Latino/Hispanic, 27% identified as Asian, 13% identified as White/Caucasian, 7% identified as Middle Eastern, 3% identified as Black/African American, and 3% identified as Multiracial. Although there was not enough heterogeneity among raters to capture systematic differences in ratings by gender and race per target photo, interrater reliability was assessed using intraclass correlation coefficients and revealed a high level of agreement between raters (0.98; *p* < .001).

#### Experiences with discrimination

The survey presented four items for both peer and institutional discrimination, drawing from an adapted version of the Adolescent Discrimination Distress Index (Fisher et al., 2000) to assess how often adolescents experienced discrimination either from peers (e.g., “Were you threatened by other kids because of your race/ethnicity”; four items; α = .75) or from societal institutions (e.g., “Were you hassled by the police because of your race/ethnicity”; four items; *α* = .85). When answering these items, students were asked to keep the ethnic‐racial group they “feel most a part of” (their forced‐choice response) in mind. The subscales were adapted to accommodate Likert scale responses (1 = *Never* to 5 = *A whole lot*) as opposed to the original dichotomous responses. Higher mean scores for these items indicate greater frequency of perceived discrimination.

#### Family ethnic‐racial socialization

The survey presented a total of 12 items measuring family ethnic‐racial socialization (ERS) drawn from the Familial Ethnic Socialization Measure (Umaña‐Taylor et al., 2004). Five items measure overt socialization, or explicit discussion regarding one's race/ethnicity (e.g., “My family teaches me about the history of my ethnic/cultural background;” *α* = .92). The other seven items measure covert socialization, or more passive experiences that socialize race/ethnicity (e.g., “Our home is decorated with things that reflect our ethnic/cultural background;” *α* = .85). Higher mean scores indicate higher levels of overt and covert ERS, respectively. The overt and covert subscales have been found to be reliable with samples from diverse ethnic backgrounds as well, asserting its validity (Umaña‐Taylor et al., 2006). Students were asked to “think about the ethnic group that you feel most a part of” when filling out these survey items.

#### Racial composition of friend groups

A section of the survey was dedicated to collecting information about the number and quality of participants' friendships. Students were asked to name up to 10 of their closest friends within the school. Friendship nominations were then matched with the friend's self‐reported race data from their respective survey to create new variables representing the proportion of a student's friends from each ethnic‐racial category.^5^

#### Demographic variables

Our analyses adjusted for the following variables: gender, school site, grade level, and immigration generation status (summarized in Table 3). The control variable for immigration generation status was created using students' reports of whether they, their parents, and/or their grandparents were born in the U.S., on U.S. territories, on U.S. military bases, or abroad. If a student was born abroad, they were classified as a first‐generation immigrant; if at least one parent was born abroad they were classified as second generation; if at least one grandparent was born abroad, they were classified as third generation; and if no one was born abroad, they were classified as fourth‐plus generation.

**TABLE 3:** Summary statistics and correlations for Biracial youth with complete cases.

Independent variables	M	SD	(1)	(2)	(3)	(4)	(5)	(6)	(7)	(8)	(9)	(10)	(11)
1. Peer discrimination	1.637	0.733	1.000										
2. Institutional discrimination	1.543	0.793	0.689	1.000									
3. Overt family ethnic‐racial socialization	2.763	1.010	0.201	0.263	1.000								
4. Covert family ethnic‐racial socialization	2.672	0.949	0.276	0.324	0.781	1.000							
5. Skin color	3.426	1.495	0.158	0.344	0.241	0.171	1.000						
6. Proportion of American Indian/Native American Friends	0.022	0.094	0.005	−0.019	0.052	0.011	−0.016	1.000					
7. Proportion of Asian friends	0.076	0.187	−0.129	−0.166	−0.012	−0.031	−0.114	−0.050	1.000				
8. Proportion of Black friends	0.236	0.337	0.120	0.207	0.159	0.137	0.396	−0.028	−0.182	1.000			
9. Proportion of Latinx friends	0.182	0.284	0.074	0.034	0.039	0.102	−0.068	−0.044	−0.173	−0.174	1.000		
10. Proportion of White friends	0.305	0.351	−0.148	−0.182	−0.137	−0.149	−0.298	−0.079	−0.028	−0.393	−0.257	1.000	
11. Proportion of multiracial	0.023	0.097	0.013	0.007	0.011	0.018	−0.021	−0.011	−0.020	−0.077	−0.024	−0.066	1.000

Abbreviations: M, mean; SD, standard deviation.

### Analysis strategy

Given the proposed research questions, logistic regression models were used to assess how our primary variables of interest (overt and covert ERS, proportion of same‐race friends, institutional and peer discrimination, and skin color) related to the racial category selections students made when encountering a monoracial paradigm of race (a forced choice survey that asked Multiracial respondents to choose just one of their ethnic‐racial backgrounds).^6^ To start, we estimated a logistic regression model that included the entire sample of Biracial students to examine Biracial youths' intentional assertion of a Biracial identity (i.e., a border identity, or refusal to select just one option in the forced‐choice question).

Subsequent logistic regression models were estimated for each specific ethnic‐racial reference group. That is, five separate models examined the selection of Black identity among Biracial Black youth, Latinx identity among Biracial Latinx youth, Native American identity among Biracial Native American youth, Asian identity among Biracial Asian American youth, and White identity among Biracial White youth, respectively. The models for the forced choice selection of Black, Latinx, Native American, Asian, and White identification included ERS, experiences of discrimination, skin color rating, proportion of friends in the focal forced choice category, and control variables. In this study, the model assessing each specific Biracial identity included only the proportion of friends of that identity as a predictor. For example, the model that included all Biracial Black students included the proportion of friends who are Black as a predictor; the model including Biracial Native American students included the proportion of friends who are Native American friends, and so on. Additionally, we estimated each of these models a second time in order to examine differences between specific Biracial combinations. In order to do this, we restricted the sample to include Biracial students who only selected a combination of the Black, Latinx, Asian, and White category options (the most frequently selected categories). The subgroups of Biracial Native American, other, Middle Eastern, and Pacific Islander groups were too small to produce estimations in most models. However, the Biracial‐White model also included Native American‐White students because that was the only Biracial‐Native American subgroup that was large enough to estimate. Indicator variables in these follow‐up models allowed us to explore any differences across specific Biracial combinations. Finally, error terms clustered at the school level were included in all models to control for fixed effects given the two school contexts represented in the dataset.

The advantage of our analytic approach is that it allows us to find commonalities within groups of Biracial students (i.e., what might all Biracial White youth have in common?) while also revealing differences between Biracial groups (i.e., what are some differences between Biracial Black and Biracial Asian students?). This approach necessarily means that most participants were included in exactly two models (i.e., a Biracial Black‐Asian individual was included in both the Biracial Asian: Asian forced choice and Biracial Black: Black forced choice models).^7^ Although this is generally uncommon in the literature as it may result in correlated error terms across the models, we did so for two reasons. First, this analytical approach allows us to identify effects that transcend youth of different Biracial backgrounds and garner insights into within‐group differences. Second, this approach addresses a longstanding issue in the area of Multiracial research by honoring the multiplicity of youth's identities. Historically, scholars have omitted Multiracial persons from analyses or lumped them into one all‐encompassing “other” category, essentially erasing the complex lived experiences of the population. Researchers motivated to recognize Multiracial realities and achieve equity through quantitative methods must think creatively and question statistical practices and norms that were developed within a “colorblind” framework (Castillo & Gilborn, 2022). Traditional analytical designs struggle to represent the complexities of Multiraciality, but by leveraging approaches such as the ones used in this paper, we make the case for developing new practices that provide insight into Multiracial realities. Our analysis provides new insight that is unavailable through alternative models and thus is worth the unconventionality.

## RESULTS

### All Biracial students

The first logistic regression model assessed whether Biracial students refused to select just one option in the forced‐choice question and therefore asserted a Biracial, or *Border*, identity (see Table 4, Column A). This model only included Biracial students who had valid data for all independent variables (*n* = 687). Recall that this analysis was largely exploratory, and no predetermined hypotheses were made.

**TABLE 4:** Logistic regression odds ratios across all models.

Independent variables	Dependent variable: Forced choice selection					
	Biracial^a^ (A)^b^	Native American (B)	Asian (C)	Black (D)	Latinx (E)	White (F)
Sample size	687^c^	145	131	261	330	472
Covert ERS	0.651***	1.436**	1.433***	1.652	2.168***	0.436***
Overt ERS	2.046***	0.717***	0.805***	0.880	0.647*	1.083
Proportion of friends from focal category	0.237	21.824**	3.239**	6.056***	4.704***	4.751***
Skin color	0.955	0.749*	1.171**	1.756*	0.742***	0.587*
Institutional discrimination	0.868	1.032	0.542*	1.518	1.616***	0.425***
Peer discrimination	0.802	0.421***	1.060	0.817**	1.025***	1.519
Boy	0.633*	0.893	1.269	2.248***	1.051	1.503**
Gender Queer	—	—	1.263	—	—	2.528
Grade	1.163	0.852	1.375***	0.777	0.797***	1.115
2nd generation	0.136***	—	0.466	3.487***	4.563	1.926
3rd generation	0.141***	1.286	0.147	4.422***	3.070	3.130*
4th generation	0.084***	1.689***	0.466***	16.396***	2.956	2.060
Constant	0.132*	7.020	0.037**	0.030*	0.726	2.607

^a^ Outcome = refused forced choice and asserted a Biracial identity.

^b^ The letters included in the column header are only to aid with directing readers to the appropriate model discussed in the main text.

^c^ While 697 Biracial students had complete cases, all 10 of the genderqueer students chose not to assert a Biracial identity. Because they perfectly predicted the outcome, they were dropped from this model.

***p < .001, **p < .01, *p < .05.

In this model, overt and covert ERS were significantly associated with the likelihood of asserting a Biracial identity while confronting a monoracial paradigm but did so in opposite directions. Having received more overt familial ERS was associated with an increased likelihood that a participant would assert their border identity and refuse the forced‐choice question (*p* < .001). However, having been exposed to more covert familial ERS was associated with a decreased likelihood that the participant would assert a Biracial identity (*p* < .001). In this model, boys were significantly less likely to assert a border identity (*p* < .05). Youth with second, third, and fourth immigrant generation status were more likely than first‐generation immigrants to select one category in the forced‐choice situation (*p* < .001). The proportion of Multiracial friends, skin color rating, peer and institutional discrimination, and grade level were not significantly associated with the likelihood of asserting a border Biracial identity.

### Biracial Native American students

In examining Biracial Native American students (*n* = 145; Table 4, Column B), contrary to our hypothesis, higher levels of overt ERS were associated with a decreased likelihood that students would select Native American as their racial category when forced to choose (*p* < .001). Yet, high levels of covert ERS were associated with an increased likelihood of selecting Native American, as hypothesized (*p* < .01). The proportion of same‐race friends was significant and in line with our hypotheses, such that having higher proportions of Native American friends predicted a higher probability that a student would select Native American as their forced‐choice response (*p* < .01). Furthermore, contrary to our predictions, Biracial Native American students with lighter skin tones were significantly more likely to select Native American when forced to choose (*p* < .05). In addition, peer discrimination was significant such that higher levels of peer discrimination were associated with a lower probability that a Biracial Native American student would select Native American as their forced‐choice response (*p* < .001). Finally, fourth‐plus generation immigrants were significantly more likely to select Native American as their racial category relative to first‐generation immigrant students (*p* < .001). No other significant associations were found.

### Biracial Asian^8^ students

The model with Biracial Asian students (*n* = 131) analyzed which variables were significant in predicting whether Biracial Asian students would select Asian as their racial category when asked to choose just one ethnic‐racial label (see Table 4, Column C). Like the Biracial Native American model, overt and covert ERS were significant but again in different directions such that higher levels of overt ERS were related to a decreased likelihood that the student would select Asian as their racial category, yet higher levels of covert ERS predicted greater a likelihood of selecting Asian as their racial category (*p* < .001). In line with our hypotheses, skin color was significant such that Biracial Asian students with darker skin were significantly more likely to self‐categorize as Asian when forced to choose. Further, institutional discrimination was significant such that higher levels of institutional discrimination were related to a decreased likelihood that a Biracial Asian student would select Asian as their forced‐choice response (*p* < .05). Again, this is contrary to our hypotheses, as we predicted that higher levels of both overt ERS and institutional discrimination would be a “pull factor” that would influence the student in selecting the Asian ethnic‐racial category. Biracial Asian students with a greater proportion of Asian friends were more likely to select Asian as their racial category (*p* < .01). Contrary to our hypotheses, peer discrimination was not significant. Regarding the control variables, fourth‐generation immigrants were less likely to select Asian as their forced choice response (*p* < .001). Further, older students were more likely to self‐categorize as Asian (*p* < .001). No significant differences were found by gender. The follow‐up logistic regression model including only Asian‐White, Asian‐Black, and Asian‐Latinx students, did not reveal any significant within‐group differences (*n* = 126; see Table 5, Column A). However, when adding indicator variables for each Biracial Asian subgroup, skin color and grade level were no longer significant. All other significant findings remained.

**TABLE 5:** Logistic regression odds ratios across all models with subgroup differences.

Independent variables	Dependent variable: Forced choice selection				
	Asian (A)^a^	Black (B)	Latinx (C)	White^b^ (D)	White^c^ (E)
Sample size	126	197	278	441	441
Covert ERS	1.497***	1.983***	2.150***	0.442***	0.442***
Overt ERS	0.656***	0.954	0.731	1.080	1.080
Proportion of Friends from focal category	3.954**	9.133***	4.399***	4.550***	4.550***
Skin color	1.059	1.883	0.921***	0.706	0.706
Institutional discrimination	0.592	1.458	2.099***	0.443***	0.443***
Peer discrimination	0.988	0.783	0.969	1.427	1.427
Boy	0.979	2.241**	1.154	1.282***	1.282***
Gender Queer	1.112	—	—	1.511	1.511
Grade	1.328	.678*	.783***	1.115	1.115
2nd generation immigrant	0.596	4.257**	6.707*	2.141	2.141
3rd generation immigrant	0.209	10.044***	4.485	3.643	3.643
4th generation immigrant	0.050***	22.506***	5.878	2.599	2.599
Biracial Native American				RG	4.888***
Biracial Asian		RG	2.523	0.457***	2.235***
Biracial Black	0.750		0.178***	0.205***	RG
Biracial Latinx	1.000	0.551***		0.425	2.078
Biracial White	RG	0.844	RG		
Constant	0.088	0.046**	0.176*	3.629	0.742

*Note*: Cells with a dash indicate that there were not enough observations to produce estimates for that variable, whereas empty cells indicate that these variables were not included in the respective models.

Abbreviation: RG, reference group.

^a^ The letters included in the column header are only to aid with directing readers to the appropriate model discussed in the main text.

^b^ Assessing hyperdescent with Biracial Native American‐White students as the reference group.

^c^ Assessing hypodescent with Biracial Black‐White students as the reference group.

***p < .001, **p < .01, *p < .05.

### Biracial Black students

The model with Biracial Black students (*n* = 261; see Table 4, Column D) assessed which variables were significant in predicting whether a Biracial Black student would select a Black identity when confronted with a monoracial paradigm of race. In this model, the proportion of same‐race friends was significant in that a higher proportion of Black friends was related to a greater likelihood of selecting the Black identity category (*p* < .001). As predicted, darker skin color was associated with a greater likelihood of selecting Black (*p* < .05). Yet, higher levels of peer discrimination were associated with a decreased odds of selecting Black as one's racial category (*p* < .01). Gender was significant in that Biracial Black boys were more likely to select Black as their forced‐choice response in comparison to Biracial Black girls (*p* < .001). Additionally, Biracial Black students that were second, third, or fourth‐generation immigrants were more likely to select Black as their forced‐choice response (*p* < .001). Contrary to our hypotheses, covert ERS, overt ERS, and institutional discrimination were not significant predictors. Additionally, grade level was not significant.

The follow‐up logistic regression model, including only Biracial Black‐White, Black‐Latinx, and Black‐Asian students (*n* = 197; see Table 5, Column B), revealed that Biracial Black‐Latinx students were significantly less likely to select Black as their racial category compared to Black‐Asian students (*p* < .001). There were no differences between Black‐White and Black‐Asian students. When controlling for specific Biracial groups, some of the independent variables demonstrated different associations than in the aforementioned overall Biracial Black model. For instance, in this follow‐up model accounting for specific Biracial Black subgroups, higher levels of covert ERS were associated with a greater likelihood of selecting Black (*p* < .001), and skin color and peer discrimination were no longer significantly associated with the outcome. Additionally, in this model, older students were significantly less likely to select Black as their forced choice response (*p* < .05).

### Biracial Latinx students

The model with Biracial Latinx students (*n* = 330; see Table 4, Column E) assessed which variables were significant in predicting the selection of Latinx for one's ethnic‐racial category. Again, in this model we saw significant associations for both covert and overt ERS but in different directions, such that more overt ERS was associated with decreased odds of selecting the Latinx category (*p* < .05), whereas more covert ERS was associated with increased odds of selecting the Latinx category (*p* < .001). Contrary to our hypothesis, skin color was significant and negative in this model such that Biracial Latinx students with lighter skin were more likely to select Latinx as their forced‐choice response (*p* < .001). Consistent with our hypotheses, both peer and institutional discrimination were significant and positive such that higher levels of discrimination were associated with self‐categorizing as Latinx (*p* < .001). Furthermore, the proportion of same‐race friends was significant, as a greater proportion of Latinx friends corresponded to a greater likelihood of selecting Latinx as the forced‐choice response (*p* < .001). Additionally, older students were significantly less likely to self‐categorize as Latinx (*p* < .001). Immigrant generation and grade level were not significantly related to self‐categorizing as Latinx.

In the follow‐up logistic regression model including only Biracial Latinx‐White, Latinx‐Asian, and Latinx‐Black students (*n* = 278; see Table 5, Column C), we found that Black‐Latinx students were significantly less likely to self‐categorize as Latinx compared to Latinx‐White students (*p* < .001). There was no difference in selecting Latinx as one's racial category between Asian‐Latinx and White‐Latinx Biracial students. When controlling for these within‐group differences, overt ERS, peer discrimination, and gender were no longer significant. We also found that 2nd‐generation immigrants were more likely to select Latinx as their racial category compared to 1st‐generation immigrants (*p* < .05).

### Biracial White students

The model with Biracial White students (*n* = 472) assessed which variables were significant in predicting whether Biracial White students would select White as their forced‐choice response. The following findings were consistent with our hypotheses. Biracial White students with a higher proportion of White friends were more likely to self‐categorize as White (*p* < .001). Higher levels of institutional discrimination were associated with a lower likelihood that a Biracial White student would select White as their racial category (*p* < .001). Biracial White students with darker skin were significantly less likely to self‐categorize as White (*p* < .05). Higher levels of covert ERS corresponded to a decreased likelihood that a Biracial White student would select White as their forced‐choice response (*p* < .001). Overt ERS and peer discrimination did not yield significant coefficients. Additionally, Biracial White boys were more likely to self‐categorize as White in comparison to Biracial White girls (*p* < .01). Finally, third‐generation immigrants were more likely to select White as their forced choice response relative to first‐generation immigrant students (*p* < .05). Grade level was not a significant predictor.

The follow‐up model, including Biracial Asian‐White, Black‐White, Latinx‐White, and Native American‐White students, allows us to assess the presence of hypo‐ and hyperdescent and thus proximity to whiteness in our sample (*n* = 441; see Table 5, Columns D & E). When testing for *hyperdescent*, we specified Biracial Native American‐White youth as the reference group. We found that Biracial Black‐White and Biracial Asian‐White students were significantly less likely to select White as their forced‐choice response in comparison to Biracial Native American‐White students, which aligns with our hyperdescent hypothesis (*p* < .001; see Table 5, Column D). Furthermore, when testing for *hypodescent*, we specified Biracial Black‐White youth as the reference group and found that Biracial Native American‐White and Asian‐White students were significantly more likely to select White as their racial category in a forced‐choice context compared to Biracial Black‐White students (*p* < .001; see Table 5, Column E). This aligns with our hypodescent hypothesis. There were no differences between Latinx‐White youth and the other Biracial White groups. In this model, skin color and immigration status were no longer significant. All other independent variables retained their significance in this follow‐up model.

## DISCUSSION

In this study, we sought to elucidate the role of contextual and individual characteristics in predicting how Biracial youth would choose to self‐categorize when confronted with a monoracial paradigm of race. Following from previous literature on Multiracial racialization, we examined a key aspect of phenotype (skin color), experiences of discrimination, familial ethnic‐racial socialization, and racial composition of friendships. Below, we discuss similarities and differences in each of these areas across different subgroups of Biracial youth in turn.

### Skin color (and proximity to whiteness)

Five of our six models revealed skin color as a significant predictor of self‐selected racial category. Biracial Black and Biracial Asian students with darker skin were more likely to select Black or Asian as their respective forced choice responses, whereas Biracial Latinx, Native American, and White students who had darker skin were less likely to self‐categorize as Latinx, Native American, and White, respectively. For Biracial Latinx youth this finding can be explained by looking at the specific within‐group differences in concert with the findings from the Biracial Black model. We know that 19% of the Biracial Latinx sample comprised Biracial Latinx‐Black students, and that they were significantly less likely to select Latinx compared to Latinx‐White youth. Furthermore, in the Biracial Black model (i.e., any student who selected Black as one of their identities), students with darker skin tones were more likely to select Black as their forced‐choice response. Biracial Latinx‐Black students likely have darker skin tones than Latinx‐White youth, and were selecting Black instead of Latinx as their racial category. Although there were not enough observations to explore within‐group differences for Biracial Native American students, we believe that the relationship between darker skin tones and a lower likelihood of selecting Native American may be due to the same reason. We do know that the largest group of Biracial Native American students in the sample was Native American and Black (41%), presumably resulting in darker skin tones for these students, which would serve as a “pull factor” towards selecting the Black racial category rather than the Native American category. This is in line with literature highlighting that Biracial Black individuals are more likely to have darker skin and other phenotypic features that result in others identifying them as Black (Albuja, Gaither, et al., 2019; Albuja, Sanchez, et al., 2019; Bradshaw, 1992; Brown, 1997; Gaither, 2015; Ho et al., 2011; Thekkedam, 2013), thus influencing the individual's choice to self‐categorize as Black.

Notably, when controlling for within‐group differences in both the Biracial Black and Biracial Asian models, the effect of skin color is no longer significant; thus, the variation in skin color scores seems to have been explained by the specific Biracial combinations of Black‐Asian, Black‐Latinx, Black‐White, Asian‐Latinx, and Asian‐White students. The same is true for the Biracial White model; before controlling for within‐group differences, we saw that skin color was significant, as predicted, such that lighter skin tones were associated with a greater likelihood of self‐categorizing as White. However, this effect becomes non‐significant once indicator variables for each Biracial group are added to the model. The significant within‐group differences for Biracial White youth gave us insight into proximity to whiteness and the presence of hyper‐ and hypodescent.

Biracial Black‐White youth had the least proximity to whiteness in that these students were significantly less likely to select White as their forced choice response relative to Asian‐White and Native American‐White students. This phenomenon is in line with literature on hypodescent, which posits that Biracial Black students are least likely to pass as and/or claim a monoracial White identity (Albuja, Sanchez, et al., 2019; Chen et al., 2014, 2018; Davenport et al., 2022; Gullickson & Morning, 2011; Lee & Bean, 2012; Miller, 2020; Young et al., 2021). Biracial Native American‐White youth appear to have the greatest proximity to whiteness, as they were significantly more likely to select White relative to Black‐White and Asian‐White youth. This is consistent with literature on hyperdescent, which suggests that, historically, Native Americans have been pressured to assimilate to whiteness and reject their Native American ancestry (Doyle & Kao, 2007b; Gullickson & Morning, 2011; Harris & Sim, 2002). In the present study, Biracial Native American‐White students were almost five times as likely to select White as their forced‐choice identity in comparison to Black‐White students.

Biracial Asian‐White students appear to fall somewhere in between, as they are significantly more likely to select White compared to Black‐White students but less likely to select White compared to Native American‐White youth. Additionally, we found that Biracial Asian students in our sample who had darker skin were more likely to identify as Asian. This finding is novel, as previous research has not articulated the conditions under which Biracial Asian students may choose to self‐categorize as Asian when forced to choose one in the same way as Biracial Black‐White and Biracial Native American‐White students (Albuja, Gaither, et al., 2019; Albuja, Sanchez, et al., 2019; Davenport et al., 2022; Doyle & Kao, 2007b; Gaither, 2015; Gullickson & Morning, 2011; Lee & Bean, 2012). Further, with respect to skin color, it is possible that choosing a White identity over an Asian identity in the forced‐choice response is more closely linked to other phenotypic markers, such as eye shape, in addition to skin color. In other words, proximity to whiteness among Asian youth and the ability to pass have less to do with skin color, even though it is on average the most dominant cue for external racial categorization (Albuja, Gaither, et al., 2019; Brown Jr. et al., 1998; Feliciano, 2016; Gaither et al., 2023; Pauker et al., 2018). Additionally, there were no significant differences between Latinx‐White students and either Black‐White or Native American‐White students. One reason the choices of Latinx youth in this sample might not be aligning with the hypo/hyperdescent patterns may be their school context. Over 70% of our sample were from the Southwest region of the United States, in a community with a high Latinx population. Thus, skin color might not serve as a “pull factor” to these students as they live in communities of people that look like them, so they do not stand out phenotypically relative to the minoritized Native American, Asian, and Black students in our sample. These findings expand on literature articulating the lived experiences of Biracial youth and clarify for which Biracial groups skin color may matter in monoracist situations where a forced selection of one identity is requested and which Biracial groups experience a greater proximity to, or distance from, whiteness.

### Experiences with discrimination

Our findings regarding experiences with discrimination were not as clear‐cut as hypothesized, as there were mixed findings across groups. Institutional discrimination was significant in the Biracial Latinx, Asian, and White models, but not in the Biracial Black, Native American, or full Biracial models. In addition, peer discrimination was significant in the Biracial Latinx, Native American, and Black models but not in the others.

The Biracial Asian and Biracial White models both revealed that higher levels of institutional discrimination were associated with a reduced odds that students would self‐categorize as Asian and White in their forced‐choice responses, respectively. This finding was contrary to our hypothesis regarding Biracial Asian students. However, this finding supports our hypothesis for Biracial White students, as experiences with institutional discrimination were associated with a greater likelihood of selecting the marginalized ethnic‐racial category when confronted with a monoracial paradigm. This is supported by the literature stating that Biracial White students who experience monoracial forms of discrimination may choose to self‐categorize with the imposed minoritized identity (“pull factor”; Christophe et al., 2022; Franco et al., 2016; Root, 1990). A similar pattern arose for Biracial Latinx youth in that exposure to higher levels of peer and institutional discrimination was each associated with a greater likelihood of selecting Latinx for their forced choice response. By contrast, Biracial Native American students who reported high levels of peer discrimination were less likely to select Native American as their racial category. Furthermore, when controlling for within‐group differences in the Biracial Asian and Black models, the effects of institutional and peer discrimination, respectively, were no longer significant; thus, controlling for specific Biracial combinations appeared to explain the variation in experiences with discrimination in these models.

These mixed results reveal that experiences with discrimination appear to have variable implications for self‐categorizing with a minoritized identity among Biracial adolescents. Previous literature has provided a monolithic view of Biracial/Multiracial youth by asserting that those who experience discrimination will feel a greater allegiance to the imposed minoritized identity, with the caveat that individuals who are White‐passing will not (Davenport et al., 2022; Franco et al., 2021; Miville et al., 2005; Root, 1990). The current findings challenge these notions and reveal more nuance in how discrimination informs racial categories, particularly when controlling for skin color and specific Biracial identities. There has been emerging evidence that the source of discrimination might influence the way Multiracial youth internalize these experiences. Of particular importance is the race of the perpetrator (Franco & Franco, 2016) and their relationship to the individual (i.e., family, peer, or stranger; Christophe et al., 2024; Franco et al., 2020; Rockquemore & Brunsma, 2002; Root, 1998). A critical next step would be to examine how Multiracial youth appraise and make sense of experiences of discrimination (e.g., Neblett et al., 2012) while considering characteristics of the perpetrator.

### Family ethnic‐racial socialization

There was a unique pattern in the associations of covert and overt ERS across models. In models comprising Biracial Latinx, Native American, and Asian American youth, we found that covert ERS was associated with an increased likelihood of selecting that category as their forced‐choice response, yet the converse was true for higher levels of overt familial ERS. This mismatch of the associations of overt and covert ERS was not hypothesized and has not yet been addressed in the literature. Research on Multiracial identity development generally suggests that engaging in cultural practices and understanding what it means to be a member of multiple ethnic‐racial groups is important for healthy Multiracial identity formation and development (Atkin & Jackson, 2021; Atkin, Jackson, et al., 2022; Bradshaw, 1992; Csizmadia & Atkin, 2022; Green & Bryant, 2023a, 2023b; Renn, 2000; Talbot, 2008). However, this work has not considered how ethnic‐racial socialization might operate differently within specific Biracial groups. Although these opposite ERS patterns are at first puzzling, we believe that looking at patterns across models reveals clues as to how covert and overt ERS might be operating differentially across specific Biracial groups.

Consider, for instance, the case of someone who is Biracial Latinx‐White and selects Latino as their forced choice response and reports very high covert FES, presumably oriented towards Latino culture. In the Latino model, we would expect that high FES would lead to selecting Latino; however, in the White model, this person would also reflect a high FES score, but they would not be choosing White. Thus, high FES would have a negative effect on choosing White in such cases. This occurs for everyone—a higher score in the model for the identity they chose would be countered by a lower score in the model for the identity they did not choose. As a result, the effect would be positive for whatever group(s) tend to have the highest average covert or overt FES. Accordingly, to interpret these patterns, we must examine trends across models.

When doing so, we see that whereas high levels of covert ERS were related to a greater likelihood of self‐categorizing with Latinx, Asian, Native American, and Black^9^ identities, the opposite was true for those selecting a White identity. These complementary findings reveal that Biracial White youth reported higher levels of covert socialization, on average, and they were selecting a minoritized identity rather than White. Recall that students were specifically asked to keep the ethnic‐racial group they “felt most a part of” in mind when responding to the ERS questions. To further interpret this finding, we considered that in a white supremacist society such as the U.S., youth are covertly socialized into a majority white culture every day, and youth who hold a White identity are often unaware of such (e.g., Leonardo, 2009). In this case, irrespective of the survey instructions, it may be that covert socialization was interpreted by youth as in reference to a racially minoritized group rather than in reference to whiteness. If so, those who responded with high values for the covert ERS items would be thinking of socialization experiences related to their non‐White identity, and this would explain their forced choice selection of a category other than White. Furthermore, it may be challenging to covertly socialize two races at home—to equally listen to the music of each culture, speak both languages, and spend time with both sides of the family—which may result in greater covert socialization of just one (Atkin & Yoo, 2019; Christophe et al., 2021; Green & Bryant, 2023a, 2023b; Miller, 2020). In fact, literature has shown that monoracial mothers are more likely to covertly socialize their Biracial children towards the one ethnic‐racial identity the mother holds, as they are typically the primary caregiver (Christophe et al., 2021; O'Donoghue, 2005; Rollins & Hunter, 2013). Practically speaking, a higher level of covert socialization may mean that it is happening in regard to only one ethnic‐racial group; this would explain why there was an association with the selection of a single category.

For Biracial Native American, Asian, and Latinx students, higher levels of overt ERS were related to not selecting those respective identities. However, in the full Biracial model, we saw that greater levels of overt ERS were related to refusing the forced‐choice question and thus asserting a Biracial, border identity. Thus, Biracial Native American, Asian, and Latinx students would be less likely to select those respective categories if they were not selecting a single identity at all and, instead, refusing the forced choice question altogether, thus asserting a Biracial identity. This corroborates literature on Multiracial families that suggests that families that engage in overt socialization are likely talking about all aspects of the child's ERI and are reinforcing the idea of being Multiracial rather than just one race or another (Atkin & Jackson, 2021; Csizmadia & Atkin, 2022; Green et al., 2021; Vezaldenos et al., 2023). This suggests that explicit conversations about being Multiracial may be an important foundation for informing the choice to *not* select just one of their group memberships even when confronted with a monoracial paradigm of race. It may be that for these students, their parents have had explicit conversations about the cultural values from both of their ethnic‐racial backgrounds, leading the students to feel equal affinity towards both of their ethnic‐racial identities. Overall, the present results suggest that *how* one is socialized, whether it is overtly or covertly, may relate to racial category choices and, ultimately, identity development processes for Biracial youth.

### Racial composition of friendship groups

**FIGURE 1:**
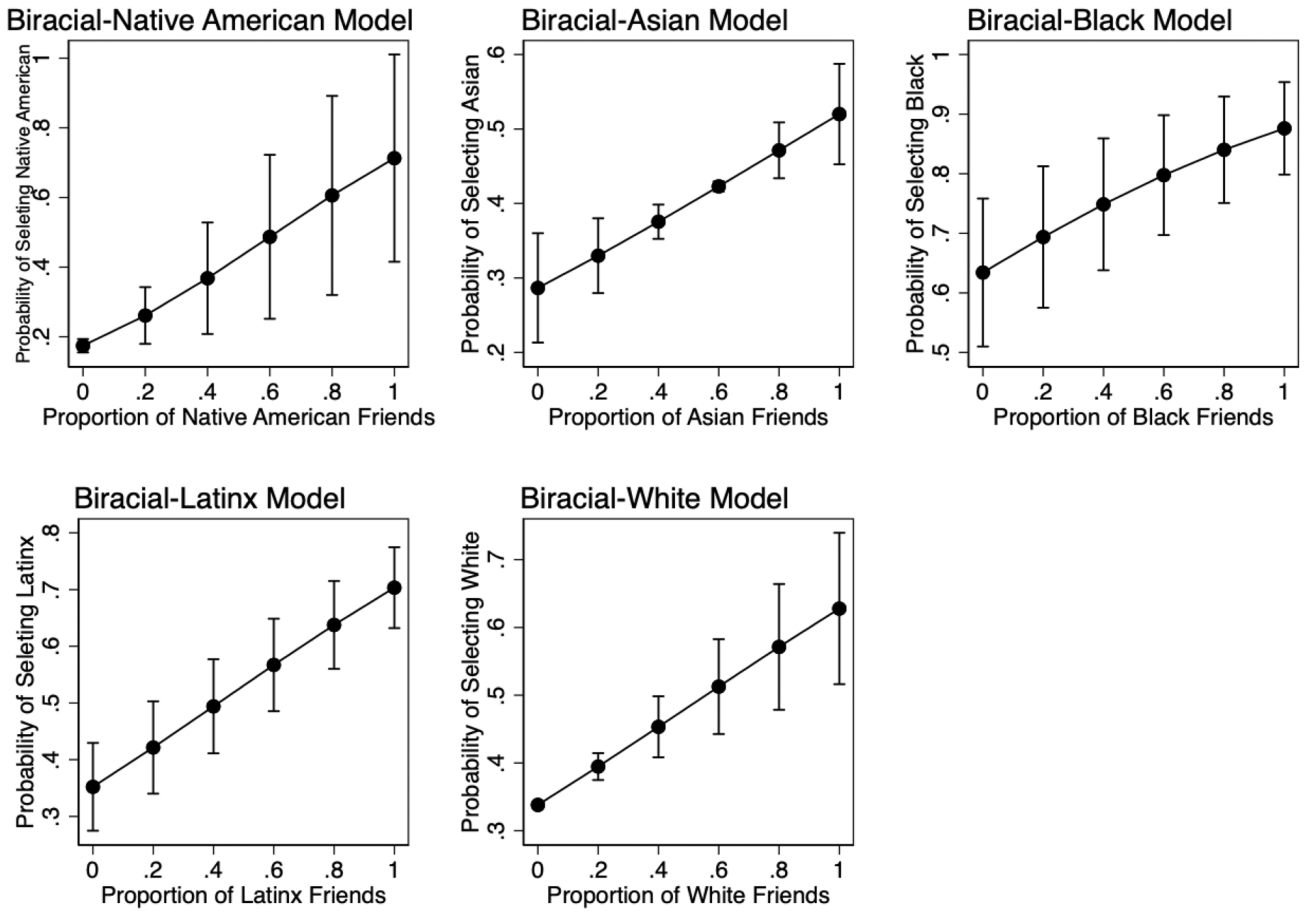
Predicted probabilities of forced choice responses by proportion of same‐race friends. Graphs show 95% confidence intervals for predicted probabilities.

One consistent finding across groups of Biracial youth is that the proportion of friends belonging to the focal forced choice category was significantly associated with selecting that category in every model. Having a greater proportion of friends who are Black, Native American, Asian, Latinx, or White was associated with selecting that group as one's racial category, respectively, with large odds ratios across all models. To unpack this relationship, post‐estimation margin commands were carried out to evaluate the predicted probabilities of selecting a particular forced‐choice response given different proportions of friends in the focal group. These analyses show that across models, students had a greater probability of selecting a particular ethnic‐racial group as their forced‐choice response as the proportion of friends from that racial group approached 1. Predicted probabilities from the ancillary analyses (see Appendix 1) are plotted in Figure 1, displaying a consistent upward trend indicating that for each model, as the proportion of same‐race friends increased, the predicted probability of selecting that ethnic‐racial group also increased.

Comparatively, Biracial‐Native American, Asian, Latinx, and White students who reported no same‐race friends had a very low predicted probabilities of selecting that ethnic‐racial identity as their forced choice response, with probabilities ranging from 0.17 to 0.35. By contrast, Biracial Native American, Asian, Black, Latinx, and White students who reported that all of their friends were the same race as them had very high predicted probabilities of selecting that ethnic racial identity as their forced choice response, with probabilities at 0.52 or greater. Biracial Black students stand out as having a higher baseline predicted probability of selecting Black. A Biracial Black student with no Black friends still has a 0.63 predicted probability of selecting Black as their forced choice response. Although this baseline predicted probability is higher than those in each of the other models, it follows the same trend such that predicted probabilities increase as the proportion of friends in the ethnic‐racial group increases. This provides further support for the role of peer groups and self‐nominated friends, in particular, in identity formation and meaning‐making processes among Biracial youth (Doyle & Kao, 2007a; Rivas‐Drake et al., 2017; Rivas‐Drake & Umaña‐Taylor, 2019; Sims, 2016).

By contrast, in the model assessing a border identity, the proportion of Multiracial friends was not significantly associated with this choice. This may be because only a small proportion of students in the sample asserted a Multiracial identity (8%) by refusing the forced‐choice question. Thus, it may be challenging for students to form friendships with youth who exclusively identify as Multiracial due to the limited opportunity pool.

### Limitations and future directions

Although these findings extend our knowledge about how Biracial youth respond to monoracist demographic forms, we recognize several limitations in this work. First, we conceptualized Biracial students in our sample as exhibiting two forms of identity: (1) a *protean identity*, meaning they shift their identity to be monoracial or Multiracial depending on the situation and context– these students were those who made a forced‐choice selection; and (2) a *border identity*, meaning they exclusively asserted a Biracial identity– these students refused the forced‐choice selection (Gabriel et al., 2023; Rockquemore, 1998; Rockquemore & Brunsma, 2002). However, we assigned these labels to students based on their cross‐sectional survey data. Without longitudinal analyses, we could not know if *protean identity* students would choose to change their racial category across time or contexts, nor could we know if *border identity* students would always assert their Biracial identity.

Additionally, students who selected two races when prompted to “select all that apply” may not in fact be Biracial, as assumed in this study. For example, students who are Afro‐Latinx may select both Latinx and Black but embody just one ethnic‐racial identity. Additionally, students who may have multiple racial identities may not see themselves as Biracial and instead strongly endorse just one ethnic‐racial identity (a *singular identity*; Gabriel et al., 2023; Rockquemore, 1998; Rockquemore & Brunsma, 2002). Thus, when initially prompted to select all racial identities that apply, they may have consciously chosen just one and would have been excluded from these analyses. Furthermore, youth who are Biracial might not have full access to socialization experiences for both groups with which they identify. For example, with the current data, there was no way to account for the ethnic‐racial identities of parents and grandparents, which would provide important insight into youths' access to knowledge, history, and cultural practices of each group (Winchester et al., 2023). Finally, our analyses are correlational and cannot unequivocally convey causality or directionality.

Future work could clarify some of the mixed findings that were revealed in this study. For instance, scholars should aim to collect data not only on skin color but also on eye shape if the research goal is to understand racial identity processes among Multiracial youth with a sizable Asian American population. Additional work can also assess the differences between overt and covert ERS and peer and institutional discrimination among Biracial adolescents. Mixed‐method approaches may be particularly useful, as participants themselves may be able to clarify and further inform the interpretation of their selections as well as of statistical results. Furthermore, previous literature has emphasized the important role parents can have in shaping ethnic‐racial identity for Biracial youth (AhnAllen et al., 2006; Atkin & Yoo, 2019; Campbell, 2007; Green & Bryant, 2023a; Herman, 2001; Lorenzo‐Blanco et al., 2013; Miller, 2020; Miville et al., 2005). Therefore, future work in this area should include the collection of data from and about parents and other family members to provide more insight into how identities in Multiracial families may inform students' racial category choices and, ultimately, their identity development processes over the course of adolescence. Finally, future research should examine the directionality of associations, such as by studying longitudinal trajectories of ethnic‐racial identity exploration and resolution—and the influence of friendship networks in such—among Multiracial youth. Such studies could continue to build from the present findings to provide a richer picture as to what informs Biracial adolescent identity choice across contexts and time.

## CONCLUSION

This study examined several factors that may inform self‐selected ethnic‐racial category choices among Biracial youth when confronted with monoracist survey questions asking them to choose between their multiple ethnic‐racial heritages. We found that the racial composition of friend groups, skin color, and covert and overt family ethnic‐racial socialization are important correlates of selecting racially minoritized identities as one's sole racial category—as operationalized by the selection of such in a forced‐choice context. Unexpected findings regarding experiences with discrimination require further investigation. Nonetheless, this study takes the field a step forward as the first quantitative study to examine how Biracial adolescents draw on aspects of their lived experience to confront monoracial paradigms of race. With our large sample of Biracial adolescents from two different regions of the U.S., we were able to closely examine Biracial subgroups, ultimately providing additional nuance regarding diverse Biracial experiences.

## CONFLICT OF INTEREST STATEMENT

We have no conflicts of interest to report.

## INFORMED CONSENT

Parent/guardian consent was obtained prior to data collection with youth.

## Data Availability

Research data are not shared.

## Footnotes

^1^
We capitalize the terms Multiracial and Biracial throughout to further assert the legitimacy of this population as well as to highlight their unique, shared racialized experiences. This is consistent with literature in this area (See Atkin, Christophe, et al., 2022; Atkin & Jackson, 2021; Atkin, Jackson, et al., 2022; Franco & Carter, 2019; Harris, 2016).

^2^
We use the term Multiracial rather than Biracial here to align with literature reviewed centering Multiracial people broadly.

^3^
The authors wish to clarify that we do not endorse this survey design practice because it reinforces monoracial paradigms of race. We acknowledge that the survey design employed during data collection for this study made monoracist assumptions, which subsequently published scholarship has now identified as potentially harmful to Multiracial respondents. While this study was conceptualized to assess the experiences of diverse youth, Multiracial youth were not centered in the design. Unexpectedly, our study produced a large Multiracial sample. The experiences of these students deserve to be explored despite these issues with construct measurement. If the study team were to produce this same study today, different methodological choices would be made.

^4^
We also verified that students that selected “Other” and wrote in a response did not indicate a Multiracial identity.

^5^
If a nominated friend self‐identified as Multiracial when prompted to select all‐that‐apply, their forced‐choice response was used to determine their group membership when calculating proportions from each ethnic‐racial group. Students who refused the forced‐choice question were coded as Multiracial. Therefore, many students had a proportion of Multiracial friends in addition to the monoracial proportion scores.

^6^
Logistic regression models employed listwise deletion to account for missing data. Thus, only students with complete cases for all variables were included in analyses (*n* = 697).

^7^
Participants that were Biracial Other, Pacific Islander, or Middle Eastern were only included in the model representing the second ethnic‐racial identity they selected as the samples for these subgroups were not large enough for independent analyses.

^8^
The term Asian is used here rather than Asian American to align with the language in the administered survey.

^9^
Covert ERS was significant in the Biracial Black model that controlled for within group differences.

## APPENDIX 1 Predicted probabilities of forced choice responses by proportion of same‐race friends

**Table:**

Forced choice response	Proportion of same‐race friends					
	0	0.2	0.4	0.6	0.8	1
Native American	0.17	0.26	0.37	0.49	0.61	0.71
Asian	0.29	0.33	0.38	0.42	0.47	0.52
Black	0.63	0.69	0.75	0.80	0.84	0.88
Latinx	0.35	0.42	0.49	0.57	0.64	0.70
White	0.34	0.39	0.45	0.51	0.57	0.63

*Note*: The predicted probabilities shown here were produced from post‐estimation margin commands following the primary analyses displayed in Table 4. Therefore, the sample sizes for the Biracial groups shown here align with the sample sizes for their respective models displayed in Table 4.

## References

[jora70012-bib-0001] AhnAllen, J. M. , Suyemoto, K. L. , & Carter, A. S. (2006). Relationship between physical appearance, sense of belonging and exclusion, and racial/ethnic self‐identification among Multiracial Japanese European Americans. Cultural Diversity and Ethnic Minority Psychology, 12(4), 673–686. 10.1037/1099-9809.12.4.673 17087528

[jora70012-bib-0002] Albuja, A. F. , Gaither, S. E. , Sanchez, D. T. , Straka, B. , & Cipollina, R. (2019). Psychophysiological stress responses to Bicultural and Biracial identity denial. Journal of Social Issues, 75(4), 1165–1191. 10.1111/josi.12347

[jora70012-bib-0003] Albuja, A. F. , Sanchez, D. T. , & Gaither, S. E. (2018). Fluid racial presentation: Perceptions of contextual “passing” among biracial people. Journal of Experimental Social Psychology, 77, 132–142. 10.1016/j.jesp.2018.04.010

[jora70012-bib-0004] Albuja, A. F. , Sanchez, D. T. , & Gaither, S. E. (2019). Identity denied: Comparing American or White identity denial and psychological health outcomes among Bicultural and Biracial people. Personality and Social Psychology Bulletin, 45, 416–430. 30084303 10.1177/0146167218788553

[jora70012-bib-0005] Atkin, A. L. , Christophe, N. K. , Stein, G. L. , Gabriel, A. K. , & Lee, R. M. (2022). Race terminology in the field of psychology: Acknowledging the growing Multiracial population in the US. American Psychologist, 77(3), 381–393. 10.1037/amp0000975 35254853 PMC9316411

[jora70012-bib-0006] Atkin, A. L. , & Jackson, K. F. (2021). “Mom, You Don't Get It”: A critical examination of multiracial emerging adults' perceptions of parental support. Emerging Adulthood, 9(4), 305–319. 10.1177/2167696820914091

[jora70012-bib-0007] Atkin, A. L. , Jackson, K. F. , White, R. M. B. , & Tran, A. G. T. T. (2022). A qualitative examination of familial racial‐ethnic socialization experiences among multiracial American emerging adults. Journal of Family Psychology, 36(2), 179–190. 10.1037/fam0000918 34516156

[jora70012-bib-0008] Atkin, A. L. , & Yoo, H. C. (2019). Familial racial‐ethnic socialization of Multiracial American Youth: A systematic review of the literature with MultiCrit. Developmental Review, 53, 100869. 10.1016/j.dr.2019.100869

[jora70012-bib-0009] Bradshaw, C. K. (1992). Beauty and the beast: On racial ambiguity. In M. P. P. Root (Ed.), Racially mixed people in America (pp. 77–88). Sage Publications, Inc.

[jora70012-bib-1010] Brown, L. J. . (1997). Assimilation and the re‐racialization of immigrant bodies: A study of TIME's special issue on immigration. The Centennial Review, 41, 603–608.

[jora70012-bib-0010] Brown, T. D., Jr. , Dane, F. C. , & Durham, M. D. (1998). Perception of race and ethnicity. Journal of Social Behavior and Personality, 13(2), 295–306.

[jora70012-bib-0011] Brunsma, D. L. (2005). Interracial families and the racial identification of mixed‐race children: Evidence from the early childhood longitudinal study. Social Forces, 84, 1131–1157.

[jora70012-bib-0012] Campbell, M. (2003). Choose one: The identity choices of Multiracial Americans on forced‐choice questions . Conference Papers ‐‐ American Sociological Association, 1–20.

[jora70012-bib-0013] Campbell, M. E. (2007). Thinking outside the (black) box: Measuring black and Multiracial identification on surveys. Social Science Research, 36(3), 921–944. 10.1016/j.ssresearch.2006.07.001

[jora70012-bib-0014] Castillo, W. , & Gilborn, D. (2022). How to “QuantCrit”: Practices and questions for education data researchers and users . (EdWorkingPaper: 22–546). Retrieved from Annenberg Institute at Brown University 10.26300/v5kh-dd65

[jora70012-bib-0015] Charmaraman, L. , Woo, M. , Quach, A. , & Erkut, S. (2014). How have researchers studied multiracial populations? A content and methodological review of 20 years of research. Cultural Diversity and Ethnic Minority Psychology, 20(3), 336–352. 10.1037/a0035437 25045946 PMC4106007

[jora70012-bib-0016] Chen, J. M. , Moons, W. G. , Gaither, S. E. , Hamilton, D. L. , & Sherman, J. W. (2014). Motivation to control prejudice predicts categorization of multiracials. Personality and Social Psychology Bulletin, 40, 590–603. 24458216 10.1177/0146167213520457

[jora70012-bib-0017] Chen, J. M. , Pauker, K. , Gaither, S. E. , Hamilton, D. L. , & Sherman, J. W. (2018). Black + White = Not White: A minority bias in categorizations of Black‐White multiracials. Journal of Experimental Social Psychology, 78, 43–54. 10.1016/j.jesp.2018.05.002

[jora70012-bib-0018] Choi‐Misailidis, S. (2004). Multiracial‐heritage awareness and personal affiliation: Development and validation of a new measure to assess identity in people of mixed race descent , [Doctoral Dissertation, Fordham University]. ProQuest Information & Learning.

[jora70012-bib-0019] Christophe, N. K. , Atkin, A. L. , Stein, G. L. , Chan, M. , Abidog, C. , Gabriel, A. K. , Lee, R. M. , Wu, C. S. , Yoo, H. C. , & The LOVING Study Collaborative . (2022). Examining Multiracial pride, identity‐based challenges, and discrimination: An exploratory investigation among Biracial emerging adults. Race and Social Problems, 14(1), 22–38. 10.1007/s12552-021-09325-4 38099096 PMC10721110

[jora70012-bib-0020] Christophe, N. K. , Atkin, A. L. , Williams, C. D. , Quick, K. N. , Wu, C. S. , & LOVING Study Collaborative . (2024). Family‐based and external discrimination experienced by multiracial individuals: Links to internalizing symptoms and familial support. Journal of Family Psychology, 38(1), 48–58. 10.1037/fam0001153 37695327

[jora70012-bib-0021] Christophe, N. K. , Stein, G. L. , & The LOVING Study Collaborative . (2021). A person‐centered analysis of ethnic–racial socialization patterns and their identity correlates in multiracial college students. Cultural Diversity & Ethnic Minority Psychology, 27(3), 332–342. 10.1037/cdp0000438 33600206 PMC8298278

[jora70012-bib-0022] Csizmadia, A. (2011). The role of racial identification, social acceptance/rejection, social cognition, and racial socialization in Multiracial youth's positive development: Multiracial youth's positive development. Sociology Compass, 5(11), 995–1004. 10.1111/j.1751-9020.2011.00418.x

[jora70012-bib-0023] Csizmadia, A. , & Atkin, A. L. (2022). Supporting children and youth in Multiracial families in the United States: Racial‐ethnic socialization and familial support of multiracial experiences. Journal of Child and Family Studies, 31(3), 664–674. 10.1007/s10826-022-02265-6

[jora70012-bib-0024] Daniel, G. R. (1996). Black and White identity in the new millennium: Severing the ties that bind. In M. Root (Ed.), The Multiracial experience: Racial borders as the new frontier (pp. 121–139). Sage.

[jora70012-bib-0025] Davenport, L. D. , Iyengar, S. , & Westwood, S. J. (2022). Racial identity, group consciousness, and attitudes: A framework for assessing Multiracial self‐classification. American Journal of Political Science, 66(3), 570–586. 10.1111/ajps.12674

[jora70012-bib-0026] Does, S. , Leslie, G. J. , Bell, A. N. , Yamaguchi‐Pedroza, K. , Shalbi, K. M. , & Shih, M. (2023). The harms of racial miscategorization: Comparing multiracial individuals' well‐being in the continental U.S. versus Hawai‘i. Cultural Diversity and Ethnic Minority Psychology, 29(3), 431–445. 10.1037/cdp0000489 34410752

[jora70012-bib-0027] Doyle, J. , & Kao, G. (2007a). Friendship choices of Multiracial adolescents: Racial homophily, blending, or amalgamation? Social Science Research, 36(2), 633–653. 10.1016/j.ssresearch.2006.12.001 19727415 PMC2735886

[jora70012-bib-0028] Doyle, J. M. , & Kao, G. (2007b). Are racial identities of Multiracials stable? Changing self‐identification among single and multiple race individuals. Social Psychology Quarterly, 70(4), 405–423. 10.1177/019027250707000409 19823596 PMC2759722

[jora70012-bib-0030] Echols, L. , Ivanich, J. , & Graham, S. (2018). Multiracial in middle school: The influence of classmates and friends on changes in racial self‐identification. Child Development, 89(6), 2070–2080. 29178469 10.1111/cdev.13000 PMC6105562

[jora70012-bib-0031] Feliciano, C. (2016). Shades of race: How phenotype and observer characteristics shape racial classification. American Behavioral Scientist, 60(4), 390–419. 10.1177/0002764215613401

[jora70012-bib-0032] Fisher, C. B. , Wallace, S. A. , & Fenton, R. E. (2000). Discrimination Distress During Adolescence. Journal of Youth and Adolescence, 29(6), 679–695. 10.1023/A:1026455906512

[jora70012-bib-0033] Franco, M. (2019). Let the racism tell you who your friends are: The effects of racism on social connections and life‐satisfaction for Multiracial people. International Journal of Intercultural Relations, 69, 54–65. 10.1016/j.ijintrel.2018.12.005

[jora70012-bib-0034] Franco, M. , & Carter, S. (2019). Discrimination from family and substance use for multiracial individuals. Addictive Behaviors, 92, 203–207. 10.1016/j.addbeh.2019.01.008 30658257

[jora70012-bib-0035] Franco, M. , Katz, R. , Pickens, J. , & Brunsma, D. L. (2020). From my own flesh and blood: An exploratory examination of discrimination from family for Black/White Multiracial people. Qualitative Social Work, 19(2), 246–266. 10.1177/1473325018815734

[jora70012-bib-0036] Franco, M. , Toomey, T. , DeBlaere, C. , & Rice, K. (2021). Identity incongruent discrimination, racial identity, and mental health for multiracial individuals. Counseling Psychology Quarterly, 34(1), 87–108. 10.1080/09515070.2019.1663788

[jora70012-bib-0037] Franco, M. G. , & Franco, S. A. (2016). Impact of identity invalidation for Black Multiracial people: The importance of race of perpetrator. Journal of Black Psychology, 42(6), 530–548. 10.1177/0095798415604796

[jora70012-bib-0038] Franco, M. G. , Katz, R. , & O'Brien, K. M. (2016). Forbidden identities: A qualitative examination of racial identity invalidation for Black/White Biracial individuals. International Journal of Intercultural Relations, 50, 96–109. 10.1016/j.ijintrel.2015.12.004

[jora70012-bib-0039] Gabriel, A. K. , Abidog, C. , Yoo, H. C. , Stein, G. L. , Christophe, N. K. , Atkin, A. , Wu, C. , & Lee, R. M. (2023). Applying critical multiracial theory to conceptualizing and measuring multiracial experiences and identity. In D. P. Witherspoon & G. L. Stein (Eds.), Diversity and developmental science (pp. 119–142). Springer International Publishing. 10.1007/978-3-031-23163-6_6

[jora70012-bib-0040] Gaither, S. E. (2015). “Mixed” results: Multiracial research and identity explorations. Current Directions in Psychological Science, 24(2), 114–119. 10.1177/0963721414558115

[jora70012-bib-1004] Gaither, S. E. , Chen, C.‐M. , Neal, S. , & Chien, S. H.‐L. (2023). Children's cross‐cultural categorizations of racially ambiguous faces in Taiwan and the U.S. Cultural Diversity & Ethnic Minority Psychology, 29(3), 385–396. 10.1037/cdp0000513 35099208

[jora70012-bib-0041] Green, M. N. , & Bryant, S. (2023a). The Multiracial‐Black socialization model: Conceptualizing racial socialization in multiracial‐Black families. Family Process, 62(3), 1075–1092. 10.1111/famp.12899 37257845

[jora70012-bib-0042] Green, M. N. , & Bryant, S. (2023b). Racially humble parenting: Exploring the link between parental racial humility and parent–child closeness in multiracial black‐white families. Race and Social Problems, 15(1), 32–44. 10.1007/s12552-023-09388-5

[jora70012-bib-0043] Green, M. N. , Charity‐Parker, B. M. , & Hope, E. C. (2021). What does it mean to be Black and White? A meta‐ethnographic review of racial socialization in Multiracial families. Journal of Family Theory & Review, 13(2), 181–201. 10.1111/jftr.12413

[jora70012-bib-0044] Gullickson, A. , & Morning, A. (2011). Choosing race: Multiracial ancestry and identification. Social Science Research, 40(2), 498–512. 10.1016/j.ssresearch.2010.12.010

[jora70012-bib-0046] Harris, J. C. (2016). Toward a critical multiracial theory in education. International Journal of Qualitative Studies in Education, 29(6), 795–813. 10.1080/09518398.2016.1162870

[jora70012-bib-1000] Harris, J. C. (2017). Multiracial college students' experiences with multiracial microaggressions. Race Ethnicity and Education, 20(4), 429–445. 10.1080/13613324.2016.1248836

[jora70012-bib-0045] Harris, D. R. , & Sim, J. J. (2002). Who is Multiracial? Assessing the complexity of lived race. American Sociological Review, 67(4), 614–627. 10.2307/3088948

[jora70012-bib-0047] Haydel, J. , Ganasi, S. , Yim, S. , Perreyclear, T. , Hernandez, L. , Zheng, H. , Jubera, K. , Pauley, T. , Gao, X. , Lin, Y. , Vera, R. , Sheng, Z. , Panichkina, A. , Markley, M. , & Willis, J. (2023). Multiracial generations: Interpersonal experiences of interminority multiracials and half‐white multiracials . 10.31234/osf.io/mh4rb

[jora70012-bib-0048] Herman, M. (2001). Forced to choose: The effects of Multiracial status on adolescent identity . Institute for Policy Research Working Paper 01–03, Northwestern University.

[jora70012-bib-0049] Herman, M. (2004). Forced to choose: Some determinants of racial identification in multi‐racial adolescents. Child Development, 75, 730–748. 15144483 10.1111/j.1467-8624.2004.00703.x

[jora70012-bib-0050] Ho, A. K. , Sidanius, J. , Levin, D. T. , & Banaji, M. R. (2011). Evidence for hypodescent and racial hierarchy in the categorization and perception of Biracial individuals. Journal of Personality and Social Psychology, 100(3), 492–506. 10.1037/a0021562 21090902

[jora70012-bib-0051] Huguley, J. P. , Wang, M. T. , Vasquez, A. C. , & Guo, J. (2019). Parental ethnic–racial socialization practices and the construction of children of color's ethnic–racial identity: A research synthesis and meta‐analysis. Psychological Bulletin, 145(5), 437–458. 30896188 10.1037/bul0000187

[jora70012-bib-0052] Jackson, K. F. , Wolven, T. , & Crudup, C. (2019). Parental ethnic racial socialization in multiracial Mexican families. Journal of Ethnic & Cultural Diversity in Social Work, 28(2), 1–26.

[jora70012-bib-0053] Johnston, M. P. , & Nadal, K. L. (2010). Multiracial microaggressions exposing monoracism in everyday life and clinical practice. In D. W. Sue (Ed.), Microaggressions and marginality: Manifestation, dynamics and impact (pp. 123–144). Wiley & Sons.

[jora70012-bib-0054] Johnston, M. P. , Ozaki, C. C. , Pizzolato, J. E. , & Chaudhari, P. (2014). Which Box(es) do i check? Investigating college students' meanings behind racial identification. Journal of Student Affairs Research and Practice, 51(1), 56–68. 10.1515/jsarp-2014-0005

[jora70012-bib-0055] Johnston‐Guerrero, M. P. , Tran, V. T. , & Combs, L. (2020). Multiracial identities and monoracism: Examining the influence of oppression. Journal of College Student Development, 61(1), 18–33. 10.1353/csd.2020.0001

[jora70012-bib-1003] Jones, C. M. , & Rogers, L. O. (2022). “There are stereotypes for everything”: Multiracial adolescents navigating racial identity under white supremacy. Social Sciences, 11(1), 19. 10.3390/socsci11010019

[jora70012-bib-0056] Kellogg, A. H. , & Liddell, D. L. (2012). “Not half but double”: Exploring critical incidents in the racial identity of multiracial college students. Journal of College Student Development, 53(4), 524–541. 10.1353/csd.2012.0054

[jora70012-bib-0057] Kich, G. K. (1992). The developmental process of asserting a biracial, bicultural identity. In M. P. P. Root (Ed.), Racially mixed people in America (pp. 304–317). Sage Publications.

[jora70012-bib-0058] Lee, J. , & Bean, F. (2004). American's changing color lines: Immigration, race/ethnicity, and multiracial identification. Annual Review of Sociology, 30, 221–242.

[jora70012-bib-0059] Lee, J. , & Bean, F. D. (2012). A postracial society or a diversity paradox? Race, immigration, and Multiraciality in the twenty‐first century. Du Bois Review: Social Science Research on Race, 9(2), 419–437. 10.1017/S1742058X12000161

[jora70012-bib-0060] Leonardo, Z. (2009). Chapter 6: The ontology of whiteness. In M. W. Apple (Ed.), Race, whiteness, and education (1st ed., pp. 91–105). Routledge. 10.4324/9780203880371

[jora70012-bib-0061] Lorenzo‐Blanco, E. I. , Bares, C. B. , & Delva, J. (2013). Parenting, family processes, relationships, and parental support in Multiracial and multiethnic families: An exploratory study of youth perceptions. Family Relations, 62(1), 125–139.

[jora70012-bib-0062] Massey, D. S. , & Martin, J. A. (2003). The NIS skin color scale. Office of Population Research, Princeton University.

[jora70012-bib-0063] Mauer, V. , Savell, S. , Davis, A. , Wilson, M. N. , Shaw, D. S. , & Lemery‐ Chalfant, K. (2020). Identification of Multiracial adolescents in research samples: An examination and critique of existing practices. Journal of Early Adolescence, 41(9), 1338–1367. 10.1177/0272431620950471

[jora70012-bib-0064] Miller, B. (2020). Biracial families: Crossing boundaries, blending cultures, and challenging racial ideologies. Journal of Family Theory & Review, 12(3), 418–424. 10.1111/jftr.12383

[jora70012-bib-0065] Miville, M. L. , Constantine, M. G. , Baysden, M. F. , & So‐Lloyd, G. (2005). Chameleon changes: An exploration of racial identity themes of Multiracial people. Journal of Counseling Psychology, 52(4), 507–516. 10.1037/0022-0167.52.4.507

[jora70012-bib-0066] Morning, A. , & Saperstein, A. (2018). The generational locus of multiraciality and its implications for racial self‐identification. The Annals of the American Academy of Political and Social Science, 677(1), 57–68. 10.1177/0002716218754774

[jora70012-bib-0067] Museus, S. D. , Sariñana, S. A. L. , Yee, A. L. , & Robinson, T. E. (2016). A qualitative analysis of Multiracial students' experiences with prejudice and discrimination in college. Journal of College Student Development, 57(6), 680–697. 10.1353/csd.2016.0068

[jora70012-bib-0068] Nadal, K. L. , Sriken, J. , Davidoff, K. C. , Wong, Y. , & McLean, K. (2013). Microaggressions within families: Experiences of Multiracial people. Family Relations, 62(1), 190–201.

[jora70012-bib-0069] Neblett, E. , Rivas‐Drake, D. , & Umaña‐Taylor, A. (2012). The promise of racial and ethnic protective factors in promoting ethnic minority youth development. Child Development Perspectives, 6(3), 295–303.

[jora70012-bib-0070] Nishina, A. , & Witkow, M. R. (2020). Why developmental researchers should care about Biracial, Multiracial, and multiethnic youth. Child Development Perspectives, 14(1), 21–27. 10.1111/cdep.12350

[jora70012-bib-0071] Norman, J. B. , Franco, M. G. , & Chen, J. M. (2023). Multiracial individuals' experiences of rejection and acceptance from different racial groups and implications for life satisfaction. The Journal of Social Psychology, 163(4), 459–479. 10.1080/00224545.2021.1996322 34843426

[jora70012-bib-0072] O'Donoghue, M. (2005). White mothers negotiating race and ethnicity in the mothering of Biracial, Black‐White adolescents. Journal of Ethnic & Cultural Diversity in Social Work, 14(3–4), 125–156. 10.1300/J051v14n03_07

[jora70012-bib-0073] Pauker, K. , Meyers, C. , Sanchez, D. T. , Gaither, S. E. , & Young, D. M. (2018). A review of multiracial malleability: Identity, categorization, and shifting racial attitudes. Social and Personality Psychology Compass, 12(6), e12392. 10.1111/spc3.12392

[jora70012-bib-0074] Perez, A. D. , & Hirschman, C. (2009). The changing racial and ethnic composition of the US population: Emerging American identities. Population and Development Review, 35(1), 1–51. 10.1111/j.1728-4457.2009.00260.x 20539823 PMC2882688

[jora70012-bib-0075] Phinney, J. S. , & Alipuria, L. L. (1996). At the interface of cultures: Multiethnic/Multiracial high school and college students. The Journal of Social Psychology, 136(2), 139–158. 10.1080/00224545.1996.9713988 8691825

[jora70012-bib-0076] Renn, K. A. (2000). Patterns of situational identity among Biracial and Multiracial college students. The Review of Higher Education, 23(4), 399–420. 10.1353/rhe.2000.0019

[jora70012-bib-0077] Rivas‐Drake, D. , Seaton, E. K. , Markstrom, C. , Quintana, S. , Syed, M. , Lee, R. M. , Schwartz, S. J. , Umaña‐Taylor, A. J. , French, S. , Yip, T. , & Ethnic and Racial Identity in the 21st Century Study Group . (2014). Ethnic and racial identity in adolescence: Implications for psychosocial, academic, and health outcomes. Child Development, 85(1), 40–57. 24490891 10.1111/cdev.12200 PMC6673646

[jora70012-bib-0078] Rivas‐Drake, D. , Umaña‐Taylor, A. , Schaefer, D. , & Medina, M. (2017). Ethnic‐racial identity and friendships in early adolescence. Child Development, 88(3), 710–724. 28322437 10.1111/cdev.12790

[jora70012-bib-0079] Rivas‐Drake, D. , & Umaña‐Taylor, A. J. (2019). Below the surface: Talking with teens about race, ethnicity, and identity. Princeton University Press. 10.2307/j.ctvc77kmw

[jora70012-bib-0080] Rockquemore, K. A. (1998). Between black and White exploring the “Biracial” experience. Race and society, 1(2), 197–212. 10.1016/S1090-9524(99)80044-8

[jora70012-bib-0082] Rockquemore, K. A. , & Brunsma, D. L. (2002). Socially embedded identities: Theories, typologies, and processes of racial identity among Black/White biracials. Sociological Quarterly, 43(3), 335–356. 10.1111/j.1533-8525.2002.tb00052.x

[jora70012-bib-0083] Rockquemore, K. A. , Brunsma, D. L. , & Delgado, D. J. (2009). Racing to theory or retheorizing race? Understanding the struggle to build a multiracial identity theory. Journal of Social Issues, 65(1), 13–34. 10.1111/j.1540-4560.2008.01585.x

[jora70012-bib-0084] Rollins, A. , & Hunter, A. G. (2013). Racial socialization of Biracial youth: Maternal messages and approaches to address discrimination. Family Relations, 62(1), 140–153. 10.1111/j.1741-3729.2012.00748.x

[jora70012-bib-0085] Root, M. (1990). Resolving “other” status: Identity development of Biracial individuals. Women & Therapy, 9(1–2), 185–205. 10.1300/J015v09n01_11

[jora70012-bib-0086] Root, M. (1998). Experiences and processes affecting racial identity development: Preliminary results from the Biracial Sibling Project. Cultural Diversity and Mental Health, 4(3), 237–247. 10.1037/1099-9809.4.3.237 9713163

[jora70012-bib-0087] Root, M. (2003). Five mixed‐race identities: From relic to revolution. In L. I. Winters & H. Debose (Eds.), New faces in a changing America: Multiracial identity in the 21st century (pp. 3–20). SAGE Publications, Inc. 10.4135/9781452233840.n1

[jora70012-bib-0088] Saperstein, A. , Kizer, J. M. , & Penner, A. M. (2016). Making the most of multiple measures: Disentangling the effects of different dimensions of race in survey research. American Behavioral Scientist, 60(4), 519–537. 10.1177/0002764215613399

[jora70012-bib-0089] Sims, J. P. (2016). Reevaluation of the influence of appearance and reflected appraisals for mixed‐race identity: The role of consistent inconsistent racial perception. Sociology of Race and Ethnicity, 2(4), 569–583. 10.1177/2332649216634740

[jora70012-bib-0090] Song, D. S. , Ahmed, A. , Gilkes Borr, T. , & Antonio, A. L. (2022). Multiracials' membership and identification practices on campus: A boundary‐work approach. Race Ethnicity and Education, 1–21. 10.1080/13613324.2022.2114510

[jora70012-bib-0091] Talbot, D. M. (2008). Exploring the experiences and self‐labeling of mixed‐race individuals with two minority parents. New Directions for Student Services, 2008(123), 23–31. 10.1002/ss.283

[jora70012-bib-0092] Thekkedam, S. C. (2013). The impact of phenotype, others' categorizations, and gender on identity outcomes in a Biracial sample . [Ph.D., Saint Louis University] https://search.proquest.com/docview/1445883910/abstract/78AF5AEAF7AF4C03PQ/1

[jora70012-bib-1002] Townsend, S. S. M. , Fryberg, S. A. , Wilkins, C. L. , & Markus, H. R. (2012). Being mixed: Who claims a biracial identity? Cultural Diversity and Ethnic Minority Psychology, 18(1), 91–96. 10.1037/a0026845 22250901

[jora70012-bib-0093] Umaña‐Taylor, A. J. , Bhanot, R. , & Shin, N. (2006). Ethnic identity formation during adolescence: The critical role of families. Journal of Family Issues, 27, 390–414.

[jora70012-bib-0094] Umaña‐Taylor, A. J. , Quintana, S. M. , Lee, R. M. , Cross, W. E. , Rivas‐Drake, D. , Schwartz, S. J. , Syed, M. , Yip, T. , Seaton, E. , & Ethnic and Racial Identity in the 21st Century Study Group . (2014). Ethnic and racial identity during adolescence and into young adulthood: An integrated conceptualization. Child Development, 85(1), 21–39. 10.1111/cdev.12196 24490890 PMC6673642

[jora70012-bib-0095] Umaña‐Taylor, A. J. , Yazedjian, A. , & Bámaca‐Gómez, M. (2004). Developing the ethnic identity scale using eriksonian and social identity perspectives. Identity, 4(1), 9–38. 10.1207/S1532706XID0401_2

[jora70012-bib-0096] Vezaldenos, V. A. , Jacobs, L.‐A. , & Rivas‐Drake, D. (2023). Raising “Antiracist Disruptors”: Illuminating socialization practices that support antiracism in multiracial households. Race and Social Problems, 15, 79–100. 10.1007/s12552-023-09389-4 36741235 PMC9891199

[jora70012-bib-0097] Winchester, L. B. , Green, M. N. , & Jones, S. C. T. (2023). Exploring the association between parental ethnic–racial socialization and parental closeness on Black–White Biracial adolescents' choice of racial identification toward blackness. Cultural Diversity and Ethnic Minority Psychology, 29(4), 482–492. 10.1037/cdp0000601 37213170

[jora70012-bib-0098] Wright, R. , Houston, S. , Ellis, M. , Holloway, S. , & Hudson, M. (2003). Crossing racial lines: Geographies of mixed‐race partnering and multiraciality in the United States. Progress in Human Geography, 27, 457–474.

[jora70012-bib-0099] Yoo, H. C. , Jackson, K. F. , Guevarra, R. P. , Miller, M. J. , & Harrington, B. (2016). Construction and initial validation of the Multiracial Experiences Measure (MEM). Journal of Counseling Psychology, 63(2), 198–209. 10.1037/cou0000117 26460977 PMC4777648

[jora70012-bib-0100] Young, D. M. , Sanchez, D. T. , Pauker, K. , & Gaither, S. E. (2021). A meta‐analytic review of hypodescent patterns in categorizing Multiracial and racially ambiguous targets. Personality and Social Psychology Bulletin, 47, 705–727. 32791890 10.1177/0146167220941321

[jora70012-bib-0101] Zamora, T. I. , & Padilla, A. M. (2024). Making sense of conflicting messages of multiracial identity: A systematic review. Frontiers in Psychology, 15, 1307624. 10.3389/fpsyg.2024.1307624 38725948 PMC11079233

